# Effects of 21st‐century climate, land use, and disturbances on ecosystem carbon balance in California

**DOI:** 10.1111/gcb.14677

**Published:** 2019-06-24

**Authors:** Benjamin M. Sleeter, David C. Marvin, D. Richard Cameron, Paul C. Selmants, A.LeRoy Westerling, Jason Kreitler, Colin J. Daniel, Jinxun Liu, Tamara S. Wilson

**Affiliations:** ^1^ U.S. Geological Survey Seattle Washington; ^2^ The Nature Conservancy San Francisco California; ^3^ U.S. Geological Survey Menlo Park California; ^4^ University of California Merced Merced California; ^5^ U.S. Geological Survey Boise Idaho; ^6^ Apex Resource Management Solutions Ltd. Ottawa ON Canada

**Keywords:** California, carbon balance, climate change, disturbance, land use, scenarios

## Abstract

Terrestrial ecosystems are an important sink for atmospheric carbon dioxide (CO_2_), sequestering ~30% of annual anthropogenic emissions and slowing the rise of atmospheric CO_2_. However, the future direction and magnitude of the land sink is highly uncertain. We examined how historical and projected changes in climate, land use, and ecosystem disturbances affect the carbon balance of terrestrial ecosystems in California over the period 2001–2100. We modeled 32 unique scenarios, spanning 4 land use and 2 radiative forcing scenarios as simulated by four global climate models. Between 2001 and 2015, carbon storage in California's terrestrial ecosystems declined by −188.4 Tg C, with a mean annual flux ranging from a source of −89.8 Tg C/year to a sink of 60.1 Tg C/year. The large variability in the magnitude of the state's carbon source/sink was primarily attributable to interannual variability in weather and climate, which affected the rate of carbon uptake in vegetation and the rate of ecosystem respiration. Under nearly all future scenarios, carbon storage in terrestrial ecosystems was projected to decline, with an average loss of −9.4% (−432.3 Tg C) by the year 2100 from current stocks. However, uncertainty in the magnitude of carbon loss was high, with individual scenario projections ranging from −916.2 to 121.2 Tg C and was largely driven by differences in future climate conditions projected by climate models. Moving from a high to a low radiative forcing scenario reduced net ecosystem carbon loss by 21% and when combined with reductions in land‐use change (i.e., moving from a high to a low land‐use scenario), net carbon losses were reduced by 55% on average. However, reconciling large uncertainties associated with the effect of increasing atmospheric CO_2_ is needed to better constrain models used to establish baseline conditions from which ecosystem‐based climate mitigation strategies can be evaluated.

## Graphical abstract

Terrestrial ecosystems are an important sink for atmospheric carbon dioxide (CO_2_). However, the future direction and magnitude of the land sink is highly uncertain. For the state of California, we modeled 32 unique scenarios, spanning 4 land use and 2 radiative forcing scenarios as simulated by four global climate models. Under nearly all future scenarios, carbon storage in terrestrial ecosystems was projected to decline, with an average loss of −9.4% (−432.3 Tg C) by the year 2100. However, uncertainty in the magnitude of carbon loss was high, and was largely driven by differences in future climate conditions projected by climate models.

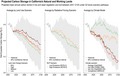

## 1 INTRODUCTION

Changes in land use have been a primary factor in the global rise of atmospheric carbon dioxide (CO_2_) and a major driver of global climate change (Le Quéré et al., 2017). Since 1850, land‐use change (LUC) has added nearly half as much carbon to the atmosphere as fossil fuel emissions and has exerted a dominant influence on the storage of carbon in terrestrial ecosystems (Houghton & Nassikas, 2017; Le Quéré et al., 2017). Changes in land use have primarily resulted in the conversion of natural ecosystems to produce food, fiber, and for establishment of settlements (Foley et al., 2005). Combined, land use and management have the potential to undermine the ability of ecosystems to produce a wide range of services (Foley et al., 2005), including the storage and sequestration of carbon to mitigate climate change (Houghton & Nassikas, 2017). Meanwhile, climate change affects ecosystem carbon balance by changing the rate of carbon uptake in vegetation (Ballantyne, Alden, Miller, Tans, & White, 2012; Ballantyne et al., 2017) and the decay and decomposition of dead organic matter (DOM) and soils (Melillo et al., 2017; Pries, Castanha, Porras, & Torn, 2017), and remains the subject of intense study (Fahey, Doherty, Hibbard, Romanou, & Taylor, 2017; Domke et al., 2018). Furthermore, studies indicate that climate change is increasing the frequency and magnitude of extreme events (Mann et al., 2017) which can alter ecosystem carbon balance by increasing gaseous emissions and through the transfer of carbon from live to DOM pools (Kurz et al., 2008). The combined effects of climate and land change can result in either positive (i.e., net emissions) or negative (i.e., net sequestration) feedbacks from the biosphere on the climate system (USGCRP, 2017). The direction and magnitude of these feedbacks will either hinder or facilitate the achievement of local to global‐scale greenhouse gas reduction targets.

Accounting for and minimizing anthropogenically linked ecosystem carbon emissions and/or maximizing the biosphere carbon sink is important for meeting the 2°C target of the Paris Agreement (Fargione et al., 2018). Despite the historically negative role in the carbon cycle, land use and management is increasingly being evaluated instead as a tool for climate mitigation (Cameron, Marvin, Remucal, & Passero, 2017; Fargione et al., 2018; Griscom et al., 2017; Houghton, Byers, & Nassikas, 2015). To evaluate the effectiveness of land‐based mitigation efforts, regional baseline estimates of carbon stocks and fluxes are needed. At management scales, spatially explicit projections spanning long temporal periods (i.e., 50–100 years) need to consider the interactive effects of changes in land use/land cover (LULC) and climate. Such projections provide a reference point against which the effects of mitigation actions, applied to different locations at different times, can be evaluated. With the rise in subnational emission reduction targets (Galarraga, de Murieta, & França, 2017) jurisdictions must work not only to mitigate emissions from both the energy and land sectors but also to understand future climate‐biosphere feedbacks and their effect on ecosystem carbon balance.

**Figure 1:**
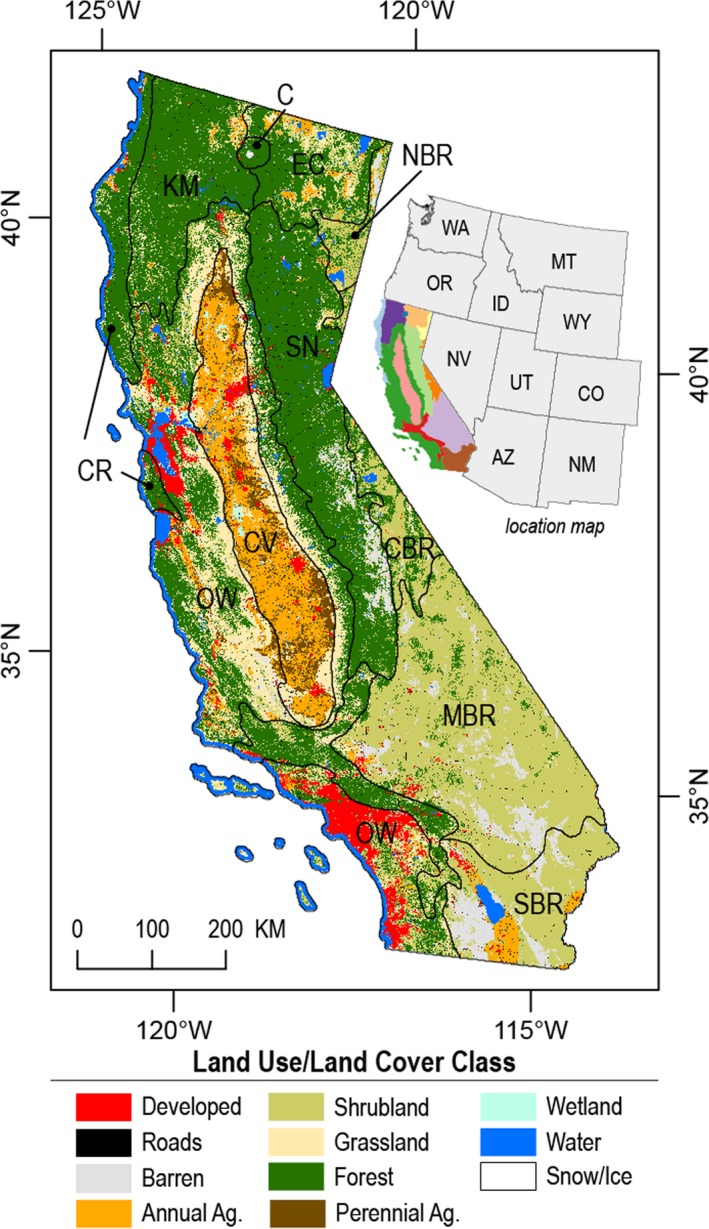
Land use/land cover map of California. Ecological regions are shown as black lines. CR is Coast Range, KM is Klamath Mountains, C is Cascades, SN is the Sierra Nevada Mountains, EC is East Cascades Slopes and Foothills, CV is Central California Valley, OW is Central and Southern California Chaparral and Oak Woodlands, SCM is Southern California Mountains, NBR is Northern Basin and Range, CBR is Central Basin and Range, MBR is Mojave Basin and Range, and SBR is Sonoran Basin and Range

The State of California exemplifies many of the challenges associated with projecting integrated effects of climatic and LUC on ecosystem carbon balance. California is a large and ecologically diverse region overlain by a complex land‐use mosaic (Figure 1). California ranks first among the United States in population, economic activity, and agricultural production value, while at the same time maintains nearly half of its land area in some form of protected status. California is a leader in subnational climate action with one of the only economy‐wide regulatory climate targets in the world (U.S. Climate Alliance, 2018). Recognizing the importance of ecosystems for emissions mitigation, the state has started to integrate ecosystem management as part of its emissions reduction strategy (California Air Resources Board, 2017). California's climate is highly variable (Swain, Langenbrunner, Neelin, & Hall, 2018), with a recent severe drought killing more than 100 million trees (Stephens et al., 2018) and a high probability of a multidecadal drought occurring this century (Cook, Ault, & Smerdon, 2015). Anthropogenic climate change is increasing the frequency of climate extremes in California (Cvijanovic et al., 2017; Diffenbaugh, Swain, & Touma, 2015), leading to changes in natural disturbance regimes (Abatzoglou & Williams, 2016; Crockett & Westerling, 2018). However, the future effects of climate and land change on ecosystem carbon balance are difficult to predict, and pose additional challenges to meeting greenhouse gas reduction targets. Furthermore, no existing studies have attempted to quantitate the effects of major controlling processes such as land use, LUC, natural disturbance, and climate change, on the carbon balance of California ecosystems.

We consider 32 unique future scenarios exploring alternative assumptions about land use and LUC, reductions in global emissions, and future climate conditions. We used a fully coupled state‐and‐transition simulation model with carbon stocks and flows to estimate changes in ecosystem carbon balance for California's natural and agricultural lands on an annual time step for the period 2001–2100 (Daniel, Sleeter, Frid, & Fortin, 2018; Sleeter et al., 2018). Scenario projections explored low, medium, high, and business‐as‐usual (BAU) LUC pathways (Sleeter et al., 2018) and were combined with climate projections for two radiative forcing scenarios (i.e., representative concentration pathway [RCP] 4.5 and RCP 8.5). RCPs were simulated by four bias‐corrected, statistically down‐scaled global climate models (GCMs) (Pierce, Cayan, & Thrasher, 2014) chosen to represent a wide range of future climate pathways in California. Finally, we integrated projections of climate‐driven drought‐induced tree mortality and wildfire, overcoming a major limitation of previous studies (Allen, Breshears, & McDowell, 2015). The goals of this study were to (a) estimate changes in California ecosystem carbon balance and their uncertainties over the recent historical past and under a range of plausible future scenarios; (b) estimate how changes are distributed across different components (i.e., carbon pools), LULC classes (e.g., forests, grasslands), and regions; and (c) develop understanding of the relative impact of major controlling processes such as land use, disturbances, and climate change.

## 2 MATERIALS AND METHODS

We used the land use and carbon scenario simulator (LUCAS), an empirical model of LUC coupled with a gain–loss model of ecosystem carbon dynamics (Selmants, Giardina, Jacobi, & Zhu, 2017; Sleeter et al., 2018; Sleeter, Sleeter, et al., 2017) to project changes in ecosystem carbon balance for the state of California under a range of climate and land‐use scenarios. The LUCAS model utilizes a fully coupled state‐and‐transition simulation model with stocks and flows (STSM‐SF; Daniel et al., 2018) to estimate changes in ecosystem carbon pools resulting from changes in land use, land cover, and disturbances (Sleeter et al., 2018). The model estimates annual changes in carbon pools resulting from vegetation productivity, litterfall, mortality, decay/decomposition, emission, leaching, and harvest. Carbon stocks and fluxes respond to changes in land use and land cover resulting from processes associated with urbanization, agricultural expansion and contraction, forest and agricultural harvest and management activities, and wildfire and drought‐induced tree mortality. The model also considers the effects of short‐ and long‐term climate variability on growth of live biomass and the turnover of DOM. Land‐use transitions in this study are based on a set of projections developed for the state of California (Sleeter, Wilson, Wilson, Sharygin, & Sherba, 2017). Carbon stocks and fluxes are based on a national assessment of ecosystem carbon balance (Sleeter et al., 2018) modified for the State of California based on methods described in Daniel et al. (2018).

### 2.1 Study area

The spatial extent of this study was the state of California covering 423,812 km^2^ (Figure 1). The State of California was subdivided into a regular grid of 1 km × 1 km simulation cells. The state type of each simulation cell was based on combinations of 12 ecological regions, 58 counties (administrative units), and 12 discrete LULC classes, including water, wetlands, snow/ice, barren, forest, grassland, shrubland, annual cropland, perennial cropland, development, and transportation/roads. For the forest, grassland, shrubland, and cropland classes we also tracked the age and time‐since‐transition (TST) of each cell as additional state variables. A general description of the structure of STSMs can be found in Daniel, Frid, Sleeter, and Fortin (2016).

### 2.2 States and transitions

**Figure 2:**
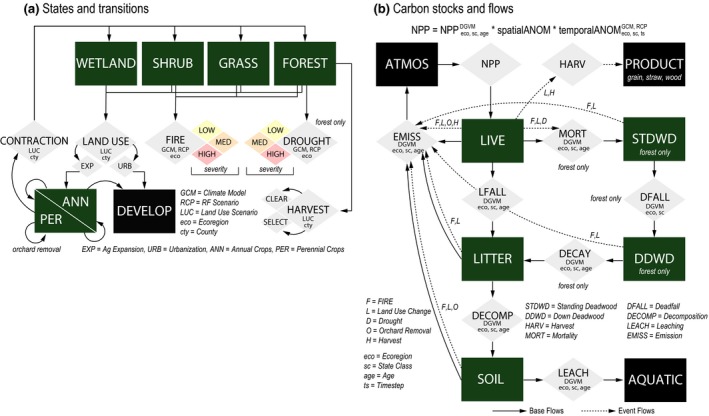
Conceptual diagram of (a) state‐and‐transition simulation model and (b) carbon stock‐flow model used in this study. Green boxes denote ecosystem state classes and carbon pools included in the estimation of ecosystem carbon storage. Gray diamonds indicate land change transition processes and carbon fluxes considered in the model. Dynamic global vegetation model with subscripts indicates that flux was parameterized with a dynamic global vegetation model as a function of the subscripts indicated. LUC, land‐use change

The STSM divides a landscape into a grid of simulation cells and then simulates the state type of each cell forward in time, as a discrete‐time stochastic process, in response to any number of possible transitions (Daniel et al., 2016). Transitions between state types were defined to represent the processes associated with urbanization, agricultural expansion and contraction, agricultural harvest and management, forest harvest, wildfire, and drought mortality (Figure 2). In total, 39 unique transition pathways were defined for this study and were based on the model described in Sleeter, Wilson, et al. (2017) and Sleeter, Sleeter, et al. (2017). The order in which transitions were applied was randomized in each time step. Several important modifications to the model were made for this study and are described below. For a thorough description of the structure of the STSM developed for California, see Sleeter, Wilson, et al. (2017); Sleeter, Sleeter, et al. (2017) and Wilson, Sleeter, and Cameron (2016).

The base land change model included a BAU and three population‐based scenarios which explored alternative urbanization pathways (Sleeter, Wilson, et al., 2017; Sleeter, Sleeter, et al., 2017). The BAU scenario was based entirely on extending historical rates of LULC change into the future and represents a future scenario pathway describing a continuation of recent historical trends. The three population‐based scenarios were originally designed to explore the effect of different rates of population growth on urban land expansion. For this study, we further modified the population‐based scenarios to also include varying rates of other anthropogenic land uses, such as agricultural expansion and contraction and forest harvest.

The ‘high’ land‐use scenario utilized a high population projection to characterize urbanization, and was further modified to explore a future with high rates of other anthropogenic land uses. Under this scenario, transition rates associated with agricultural expansion and contraction were derived by sampling, with replacement, from the period 1997–2002, which was a period of sustained growth in the areal extent of California agricultural lands (Sleeter, Wilson, et al., 2017; Sleeter, Sleeter, et al., 2017). Similarly, for transitions associated with forest harvest, we sampled, with replacement, from the 2002–2009 period. A similar approach was used to construct the ‘low’ land‐use scenario, where the rates of agricultural land use were sampled from the 1993–1996 period and transitions associated with forest harvest were sampled from the 2010–2014 period. These rates were combined with a low population projection and corresponding low rate of urbanization. For the ‘BAU’ and ‘medium’ scenarios, we sampled, with replacement, from the full time series of historical land‐use data for each transition type. As such, the only difference between the ‘BAU’ and ‘medium’ land‐use scenarios was the rate of urbanization. The ‘BAU’ scenario projected urbanization using the full time series of historical urbanization data while the ‘medium’ land‐use scenario used a medium population projection as described in Sleeter, Wilson, et al. (2017) and Sleeter, Sleeter, et al. (2017).

Forest harvest activities in California are dominated by softwoods, which make up approximately 58% of the total forest area of the state and are dominated by a mixed conifer group (includes mixed stands of Douglas fir, ponderosa pine, sugar pine, Jeffrey pine, white and red fir, incense cedar, and other true fir species), ponderosa pine, and other western softwoods (Christensen, Waddell, Stanton, & Kuegler, 2016). Hardwoods make up approximately 40% of the state's forest lands and are dominated by western oak species; however, they make up less than 1% of the total statewide harvest (McIver et al., 2015). Conversely, the combined harvest of Douglas fir, true firs, ponderosa pine, redwood, and sugar pine accounts for nearly 94% of all harvest in California (McIver et al., 2015). In the LUCAS model, forest harvest was characterized as either stand replacing (clear‐cut) or partial harvest (selection) events. Annual historical harvest rates were derived from annual historical maps of forest disturbance (U.S. Geological Survey [USGS] LandFire Program, 2014) available for the period 1999–2014. In projected years, forest harvest transitions were estimated by sampling, with replacement, from the historical time series of data, where covariance between harvest types and rates by county was preserved. The minimum age for forest harvest was set to 40 years old for clear‐cut and 20 years old for selection harvest. Cells selected for clear‐cut harvest subsequently had their age reset to 0 while cells selected for selection harvest did not experience a change in age. Spatial maps were used to prohibit harvest from protected lands (U.S. Geological Survey [USGS] Gap Analysis Program [GAP], 2016). Carbon transfer rates resulting from clear‐cuts were based on Sleeter et al. (2018). For selection harvest, we assumed 20% of live biomass pool was transferred to the harvested wood products (HWP) pool.

Wildfire and drought‐induced tree mortality were estimated based on submodels which were integrated into the LUCAS framework. An exogenous statistical fire model was used to derive burn area projections for each climate scenario (Sleeter, Wilson, et al., 2017; Sleeter, Sleeter, et al., 2017). For the projected period, burn area was estimated for each GCM and RCP based on a statistical model of wildfire which considered the effects of climate, vegetation, population density, and fire history (Westerling, 2018). For each scenario, the annual projected burn area was summarized for each ecoregion and climate scenario. The LUCAS model was then used to simulate individual fire events which spread across the landscape within a time step. Fire events were projected based on (a) the expected annual burn area within an ecoregion as provided by the exogenous statistical fire model; (b) the relative probability of an individual cell experiencing a fire; (c) the stationary distribution of fire size as calculated from historical fire maps for each ecoregion; and (d) the distribution of fire severity classes as calculated from historical fire maps for each ecoregion.

A similar approach was used to estimate the annual extent of drought‐induced tree mortality. We used the 60‐month Standardized Precipitation Evapotranspiration Index (SPEI; Vicente‐Serrano, Beguera, & Lopez‐Moreno, 2010) to track long‐term drought annually across California using PRISM (PRISM Climate Group, Oregon State University, 2016) 4‐km historical climate data for monthly temperature and precipitation inputs calibrated to aerial detection survey forest mortality maps (Moore, McAfee, & Iaccarino, 2018). We fit a binomial generalized linear model for each of the three mortality classes, using SPEI as a single predictor in the model. We used these models to estimate future drought‐induced mortality for each GCM and RCP scenario on an annual time step. We estimated the annual mortality area for each ecoregion from the model outputs and sampled, with replacement, from a Gaussian distribution created from these annual ecoregional means and an assumed 50% standard deviation. Annual relative probability maps derived from the spatial predictions were used within LUCAS to constrain the pattern of disturbance. Additional details describing the wildfire and drought‐induced tree mortality submodels can be found in the Data S1: Methods.

### 2.3 Carbon stocks and flows

With the STSM‐SF method, the fate of continuous carbon stocks can be simulated for each simulation cell, based on a suite of continuous flows (i.e., carbon fluxes) specifying the rates at which these stock levels change over time (Daniel et al., 2018). Carbon stocks considered in this study included a single live pool (living biomass), three DOM pools (standing deadwood, down deadwood, and litter), and a soil pool (SOC, soil organic carbon). In addition, we tracked carbon in three product pools, including HWP and carbon stored in agricultural products (grain and straw pools). Pools representing carbon stored in the atmosphere and aquatic pools were tracked to ensure the mass balance of carbon was maintained. For live, DOM, and soil pools, initial carbon stocks were estimated for each land cover class (forest, grasslands, shrublands, and annual and perennial agriculture) and ecoregion based on a regionally calibrated dynamic global vegetation model (DGVM; Daniel et al., 2018; Selmants et al., 2017; Sleeter, Liu, Daniel, Frid, & Zhu, 2015; Sleeter et al., 2018) and a remote sensing‐derived map of forest age (see Data S1: Methods).

The LUCAS model estimates change in carbon stocks (i.e., fluxes) resulting from growth, mortality, litterfall, deadfall (e.g., standing to down deadwood), decomposition, emission, and leaching (Figure 2). In addition, LUCAS estimates change in carbon stocks and fluxes resulting from land use, LUC, and disturbances. Average annual growth was estimated for each LULC class and ecoregion based on an empirical model of net primary production (NPP; Del Grosso et al., 2008). Ecoregion and land cover‐specific turnover rates were then used to represent the flow of carbon from live to DOM pools resulting from litterfall and mortality based on an analysis of output from a DGVM (Daniel et al., 2018; Sleeter et al., 2018). Rates of decay and decomposition of DOM, as well as the lateral flux from of carbon from terrestrial to aquatic systems (i.e., leaching), were specified for each ecoregion (Sleeter et al., 2018). An annual multiplier was applied to DOM turnover rates based on a Q10 function (Kurz et al., 2009). We assumed a Q10 of 2.0 for the decay of down deadwood, decomposition of litter, and gaseous emissions from the soil pool (Pries et al., 2017), and a Q10 of 2.65 was assumed for the litter pool. Lastly, transition‐triggered flows (e.g., flows resulting from transitions such as harvest or urbanization; Daniel et al., 2018) result in additional fluxes to product pools and the atmosphere and were derived from Sleeter et al. (2018).

### 2.4 Initial conditions

The LUCAS model was initialized with spatially explicit maps representing spatial strata, LULC type, and age (Figure S1). All maps were projected to an Albers Equal Area Conical Projection using the NAD83 Datum. Each cell had a spatial resolution of 1 km × 1 km. Three levels of spatial stratification were used in the model, including ecological regions, counties, and land ownership. First, an ecoregion map was developed based on Level III ecoregions of the conterminous United States as defined by the US Environmental Protection Agency (Omernik, 1987). Second, an administrative boundaries map was derived based on county boundaries from the US Census Bureau. Lastly, three levels of ownership were defined, including federal, nonfederal, and private, based on the US Protected Areas Database v1.4 (U.S. Geological Survey [USGS] Gap Analysis Program [GAP], 2016). Initial LULC class for each cell was based on Sleeter, Wilson, et al. (2017) and Sleeter, Sleeter, et al. (2017) with modifications for perennial croplands described in Data S1: Methods.

Initial carbon stocks for each simulation cell were estimated based on the ecoregion, LULC class, and age of each cell and an age‐to‐carbon look‐up table derived from the output of the DGVM (Daniel et al., 2018; Sleeter et al., 2018; Figure S2). Initial stock estimates were then scaled using a stationary spatial growth multiplier to reflect within‐ecoregion heterogeneity of carbon pools. The spatial multiplier was estimated using 30 year climate normal's and the empirical NPP model, where values for each cell reflected the NPP anomaly relative to each cell's ecoregion and LULC class type. The soil carbon pool was estimated from a soil carbon stock spatial layer using soil property prediction maps (see Data S1: Methods). We used this soil carbon map to scale the regional output from the DGVM following methods developed and described in Daniel et al. (2018).

### 2.5 Scenario simulations

We simulated 32 scenarios in total, spanning all combinations of 4 LULC scenarios, 2 radiative forcing scenarios (i.e., RCPs), and 4 climate models (i.e., GCMs). For each scenario we ran 100 Monte Carlo realizations of the model. The four LULC scenarios were based on those published in Sleeter, Wilson, et al. (2017) and Sleeter, Sleeter, et al. (2017) which explored alternative futures based on ‘BAU’ trends and three scenarios exploring alternative projections of population growth (low, medium, and high). Down‐scaled climate data from the Localized Construction Analogs (LOCA) dataset were used to represent future climate conditions for the RCP 4.5 and RCP 8.5 radiative forcing scenarios (Pierce et al., 2014). Climate models chosen represent ‘hot‐dry’ (HadGEM2‐ES), ‘hot‐wet’ (CNRM‐CM5), ‘average’ (CanESM2), and ‘complementary’ (MIROC5) conditions, which were the subset of global climate models selected for the California Fourth Climate Change Assessment as models meant to represent a range of possible futures for the state (Bedsworth, Anderson, Franco, Anderson, & Wilhelm, 2017; Pierce, Cayan, & Dehann, 2016). All scenario simulations were run at 1 km × 1 km spatial resolution on an annual time step for the period 2001–2101. A baseline period spanning the years 2001–2015 was defined where simulations utilized real historical LULC and climate data inputs; projections departed in 2016 based on 1 of the 32 individual future pathways. Positive values reported in this study denote a net increase in carbon storage in terrestrial ecosystems while negative values denote a net loss from ecosystems. The term ‘net ecosystem productivity’ (NEP) refers to the net difference in carbon storage resulting from plant productivity (i.e., NPP) and respiration by heterotrophic organisms (Rh). Net ecosystem carbon balance (NECB) refers to the net change in total carbon stored in ecosystems. For this study, the primary difference between NEP and NECB is the inclusion of carbon losses resulting from LUC, disturbances, harvest, and aquatic leaching in the calculation of NECB.

### 2.6 Effect of CO_2_ fertilization

Increasing atmospheric CO_2_ (*C*_a_) concentration has a direct, positive effect on carbon assimilation by plants through photosynthesis (Franks et al., 2013). However, there is a large degree of uncertainty in the persistence and magnitude of this effect, particularly over long time horizons. To explore the importance of the CO_2_ fertilization effect (CFE) on ecosystem carbon balance, we conducted a series of simulations to test the sensitivity of the model to the introduction of a CFE on NPP. We used the ‘BAU’ land‐use scenario and CanESM2 (‘average’) climate model to test a range of CFE rates for both the RCP 4.5 and RCP 8.5 scenarios. CFE rates were selected to span the range of values observed in the literature, which generally show increases in NPP ranging from 9% to 23% when *C*_a_ levels were increased by ~50% (i.e., an increase of ~180 ppm CO_2_; Norby et al., 2005; Norby, Warren, Iversen, Medlyn, & McMurtrie, 2010). The LUCAS model was parameterized with an NPP CFE multiplier based on a *β* factor representing the annual change in NPP (%) for every 100 ppm increase in *C*_a_. We tested five *β* levels, ranging from 2% to 14%, which are generally consistent with the range of observed CFEs from free air CO_2_ enrichment (FACE) experiments (5%–25% increases in NPP given exposure to an increase of ~180 ppm of CO_2_). Additionally, we introduced a third scenario where we assumed the CFE reached saturation at 600 ppm which is generally the upper limit from FACE experiments; this threshold is reached in ~2060 under the RCP 8.5 scenario. Under this scenario we assumed no additional CFE occurred despite *C*_a_ levels increasing to ~930 ppm by 2100. Thus, the NPP CFE multiplier was calculated as:

$${\text{NPP}}_{\text{CFE}}^{t,s}={\text{NPP}}_{\text{NOCFE}}^{t,s}\times \left(\frac{1+\Delta C_{a}^{t,s}\times \beta }{100}\right)$$

where ${\text{NPP}}_{\text{CFE}}^{t,s}$ is the projected NPP including CFE for year *t* (a year between 2001 and 2100) and scenario *s* (either RCP 4.5 or RCP 8.5), ${\text{NPP}}_{\text{NOCFE}}^{t,s}$ is the NPP projected by the model in the absence of CFE for year *t* and scenario *s*, $\Delta C_{a}^{t,s}$ is the difference in *C*_a_ between year *t* and the base year of 2001 for scenario *s*, and *β* is a percentage increase in NPP for a 100 ppm increase in *C*_a_.

## 3 RESULTS

### 3.1 Contemporary change in carbon stocks

**Figure 3:**
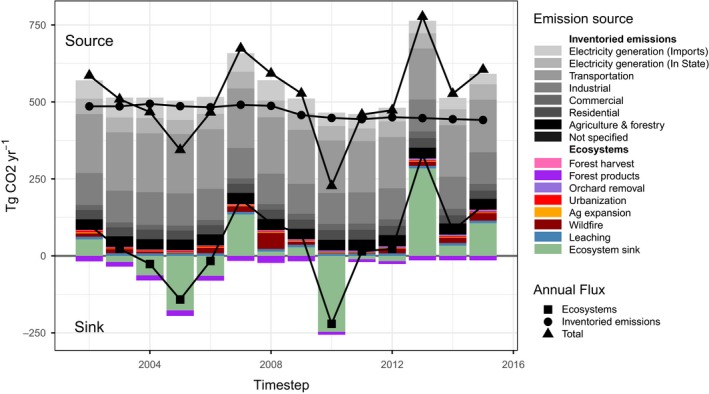
Total CO_2_ emissions and net ecosystem sink in California by sector and source for the period 2002–2015. Anthropogenic emissions are show in gray bars and ecosystem emissions and sinks are shown in colored bars. Positive values shows emissions to the atmosphere and negative values show a net sink in ecosystems. Anthropogenic emissions (gray bars) are from the California Air Resources Board

In 2001, California ecosystems stored an estimated 4,823.1 Tg C with 37.4% (1,804.3 Tg C) stored in live vegetation, 54.8% (2,643 Tg C) stored in soils, and 7.8% (375.9 Tg C) stored in DOM. Between 2001 and 2016, the total ecosystem carbon declined by −188.4 (−203.5 to −173.4) Tg C (mean and 95% Monte Carlo confidence intervals) with the mean annual source/sink rate ranging from −89.8 (source) to 60.1 Tg C/year. Between 2001 and 2011, the NECB of California ecosystems was −2.5 Tg C/year, with the carbon losses increasing 11‐fold (−40.8 Tg C/year) during the severe drought of 2012–2015 (Table 1). During this period, the cumulative change in total ecosystem carbon was −163.1 Tg C, equivalent to approximately 34% of the state's total greenhouse gas emissions over the same period (California Air Resources Board, 2018; Figure 3). Between 2001 and 2015, the mean annual NECB from California ecosystems (−13.5 Tg C/year) was equivalent to 11% of the state's average annual total greenhouse gas emissions. Table 1 shows the estimated carbon stocks and fluxes for each time step over the historical period.

**Table 1:** Annual carbon stocks and fluxes for California for the period 2001–2015. The transfer column represents the cumulative carbon transferred from ecosystem classes to nonecosystem classes (e.g., development). The LULCC column represents carbon losses due to land use, LUC, and disturbances

	Stocks				Fluxes					
Timestep	Live	DOM	Soil	TEC	NPP	Rh	NEP	LULCC	NECB	Transfer
2001	1804.3	375.9	2,643	4,823.1	—	—	—	—	—	—
2002	1782.6	371.5	2,639.9	4,794	136.2	126	10.1	37.5	−27.3	1.8
2003	1783.1	368.7	2,634.8	4,786.7	155.9	124.7	31.2	37.5	−6.3	2.9
2004	1791	373.2	2,628.6	4,792.9	165.6	121	44.6	37.3	7.2	3.9
2005	1827.5	379.5	2,623.4	4,830.4	198.2	119.1	79.1	40.5	38.6	5.1
2006	1826.3	386.4	2,621.1	4,833.8	168.6	121.1	47.5	43	4.5	6.2
2007	1775.3	387.9	2,619.7	4,782.9	108.2	121.9	−13.7	36.4	−50.1	7.1
2008	1756.1	382.2	2,614.9	4,753.3	138.5	118.6	19.9	48.7	−28.7	8.1
2009	1740.9	383	2,609.5	4,733.4	132.6	116.1	16.4	35.7	−19.2	8.7
2010	1799.3	389.2	2,604.4	4,793	209.4	109.2	100.1	40	60.1	9.4
2011	1780.7	402.3	2,605.5	4,788.4	144.4	113.4	31	35.1	−4.1	9.8
2012	1781.7	393.2	2,607	4,781.9	155.9	123.7	32.2	38.4	−6.2	10.2
2013	1698.3	387.5	2,605.4	4,691.2	63.7	121.9	−58.2	31.6	−89.8	11.2
2014	1705.5	365	2,597.3	4,667.8	142.4	127.9	14.5	37	−22.5	12.2
2015	1658.5	376.9	2,586.9	4,622.3	113.1	120.7	−7.6	37	−44.6	13.1

Abbreviations: LULCC, land use and land cover change; NECB, net ecosystem carbon balance; NEP, net ecosystem productivity; NPP, net primary production; DOM, dead organic matter; Rh, heterotrophic respiration; TEC, total ecosystem carbon.

### 3.2 Contemporary drivers of carbon stock change

Declines in ecosystem carbon were driven by a combination of factors, including interannual variability in weather and climate, changes in land use and land cover, wildfire, and drought, and represents an important positive feedback to the climate from terrestrial ecosystems. The largest drivers of ecosystem carbon loss were associated with declines in plant growth during years with poor growing conditions, gaseous emissions resulting from wildfire, and removal of carbon resulting from agricultural and forest harvest activities. Over the contemporary period, NPP averaged 145.2 Tg C/year while heterotrophic respiration (Rh) remained relatively constant at an average annual rate of 120.4 Tg C/year. A declining trend in NPP, owing primarily to sustained drought conditions over much of the state beginning in 2013, and a relatively stable rate of ecosystem respiration, resulted in NEP of 24.8 Tg C/year (Table 1). However, the effects of year‐to‐year variability in weather and climate had profound impacts on ecosystem carbon balance. For example, in 2013, statewide NPP was estimated to be reduced by 59% over the 2001–2010 average while NEP was estimated to be reduced by 256%. Moreover, since 2007, statewide NPP has been on average 15% lower than the average of the previous decade with only a single year (2010) with above average productivity (Table 1).

Carbon losses due to land use, LUC, disturbances such as fire, and lateral transfer to aquatic systems, removed an average of 38.3 Tg C/year. Table 2 shows the estimated mean annual rate of carbon flux between various pools over the historical period. The largest annual removals were associated with harvest of agricultural lands with an average annualized rate of 16.7 Tg C/year (9.8 Tg C/year in grain and 6.9 Tg C/year in straw). The removal/replanting of orchards contributed an additional loss of 0.5 Tg C/year through emissions from biomass and soils. On average, wildfire resulted in annual ecosystem carbon losses of 4 Tg C/year. However, large interannual variability in burn area resulted in annual carbon losses ranging from a low of 0.4 Tg C year in 2010 to a high of 14.4 Tg C year in 2008. Forest harvest activities, including both clear‐cut and selection harvest, represented the second largest LULC‐related flux (after harvest of agricultural products), accounting for a combined 4.3 Tg C/year being transferred from live carbon pools. Transfers from ecosystems to HWP pools accounted for 3.9 Tg C/year of the annual carbon loss, while an additional 0.4 Tg C/year was lost to the atmosphere through gaseous emissions. Other land use and LUCs, including urbanization (0.8 Tg C/year) and expansion of agriculture (0.5 Tg C/year) accounted for an additional 1.3 Tg C/year removed from terrestrial ecosystems. Other carbon losses resulted from the lateral flux from terrestrial to aquatic ecosystems (i.e., ‘Leaching’) and background emissions from biomass which were estimated at 2.3 and 9.2 Tg C/year, respectively. Cumulatively over the 2001–2015 period, LULC and disturbances resulted in 535.7 Tg C being removed from terrestrial ecosystems.

**Table 2:** Annual carbon fluxes for the historical period. Unless specified otherwise, emissions fluxes reflect the combined emissions from all ecosystem carbon pools (e.g., living biomass, litter, standing deadwood, down deadwood, and soil). Values represent the mean, minimum, and maximum annual values for the period 2001–2015

Transition group	Flow	From stock	To stock	Mean	Min	Max
Automatic flows	Growth	Atmosphere	Living biomass	145.2	63.5	209.8
	Litterfall	Living biomass	Litter	105	87.2	118.1
	Mortality	Living biomass	Standing deadwood	10.6	9.9	11
	Deadfall	Standing deadwood	Down deadwood	13.2	12.5	14.3
	Decay	Down deadwood	Litter	14.1	13.6	14.4
	Decomposition	Litter	Soil	62.2	56.2	66.5
	Emission (biomass)	Living biomass	Atmosphere	9.2	8.7	9.5
	Emission (litter)	Litter	Atmosphere	58.1	51.3	62.1
	Emission (soil)	Soil	Atmosphere	62.3	57.3	67.1
	Leaching	Soil	Aquatic	2.3	2.3	2.3
	Harvest (grain)	Living biomass	Ag products	9.8	6.4	13.9
	Harvest (straw)	Living biomass	Ag products	6.9	3.8	10
Ag expansion	Emission	All pools	Atmosphere	0.5	0.1	0.9
	Harvest	Living biomass	Wood products	0.1	0	0.2
Urbanization	Emission	All pools	Atmosphere	0.7	0.2	1.8
	Harvest	Living biomass	Wood products	0.1	0	0.4
Clear‐cut harvest	Emission	All pools	Atmosphere	0.4	0.1	0.7
	Harvest	Living biomass	Wood products	3.2	0.9	5.6
	Mortality	Living biomass	Deadwood	1.6	0.4	2.8
Selection harvest	Harvest	Living biomass	Wood products	0.7	0.1	1.3
Orchard removal	Emission	All pools	Atmosphere	0.5	0	2.2
Fire	Emission	All pools	Atmosphere	4	0.3	15.2
	Mortality	Living biomass	Deadwood	2.7	0.2	11
Drought	Mortality	Living biomass	Deadwood	3.2	0.6	17.3

In addition to the direct carbon losses (through gaseous emissions and/or transfer to product pools) discussed above, ecosystem disturbances resulted in the transfer of carbon between live and DOM pools which result in future committed emissions through decay and decomposition (Table 2). Cumulatively, wildfire accounted for 38.1 Tg C (2.7 Tg C/year) being transferred from live to DOM pools (primarily to standing deadwood), although as with gaseous emissions, interannual variability was high with annual fluxes ranging from 0.2 to 10.1 Tg C/year. Similarly, forest harvest activities resulted in the cumulative transfer of 22.4 Tg C (1.6 Tg C/year) from live to (primarily) down deadwood pools resulting from the mortality of tree roots on harvested stands. Drought mortality was the largest overall driver of increases in DOM statewide, however, the effects of drought were highly variable in space and time. Prior to 2015, drought‐induced mortality accounted for an annual transfer of 3.2 Tg C/year. However, in response to prolonged exceptional drought conditions, mortality increased to 16.9 and 29.4 Tg C/year in 2015 and 2016, respectively. As a result, the historical period (including 2016) realized a net increase in DOM pools statewide of 9.8% (36.9 Tg C).

### 3.3 Projected change in ecosystem carbon balance

**Figure 4:**
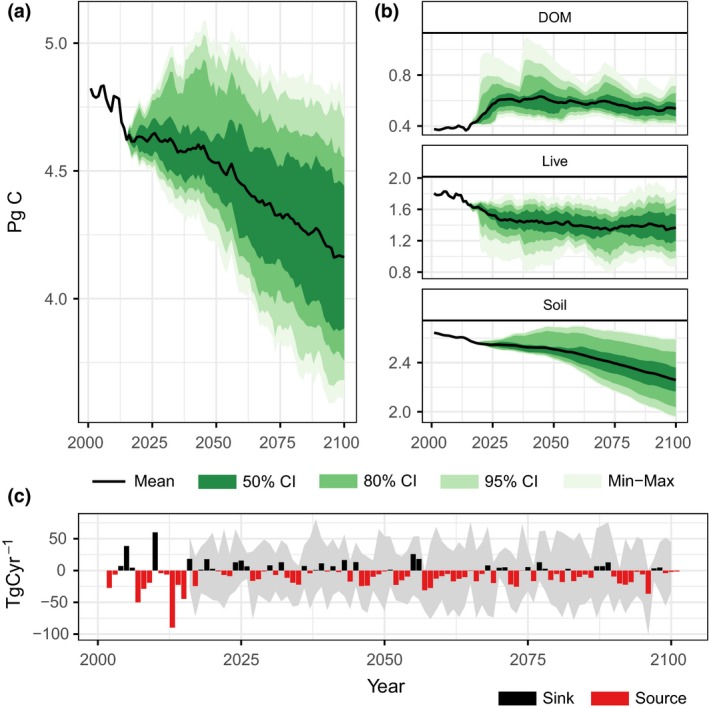
Projected changes in total ecosystem carbon (a), carbon stocks including dead organic matter, live biomass, and soil (b), and net ecosystem carbon balance (NECB; c). Mean and Monte Carlo confidence intervals were calculated across all 32 scenarios and iterations of the model. Ecosystem carbon pools include carbon stored only in ecosystem classes, including forests, grasslands, shrublands, and agriculture

This study projected changes in ecosystem carbon balance for 32 unique future pathways representing all possible combinations of two radiative forcing trajectories (RCP 4.5 and RCP 8.5) as simulated by four global climate models (CanESM2, CNRM‐CM5, HadGEM2‐ES, and MIROC5), and four land‐use scenarios (BAU, high, medium, low; Sleeter, Sleeter, et al., 2017; Sleeter, Wilson, et al., 2017). Each scenario was replicated for 100 Monte Carlo simulations. Figure 4 shows the mean projected total ecosystem carbon (Figure 4a), live, DOM, and soil carbon stocks (Figure 4b), and NECB (Figure 4c) for California over the historical and projected periods. When averaged across all scenario simulations, carbon stored in terrestrial ecosystems was projected to decline by −432.3 Tg C between 2015 and 2100, representing a loss of −9.4% from 2015 levels. An additional 68.5 Tg C was transferred from ecosystem classes (e.g., forest, grassland, shrublands, agricultural lands) to other LULC classes which were not further considered in this study (e.g., development, wetlands). The magnitude of the decline in ecosystem carbon was highly uncertain, with estimates ranging from a decline of −916.2 Tg C to a net increase of 121.2 Tg C; the mean annualized source/sink rate was projected to range from −10.8 to 1.4 Tg C/year depending on climate model, radiative forcing scenario, and land‐use scenario. Declines in ecosystem carbon were driven by declines in live and soil pools averaging −290.1 and −328.3 Tg C, respectively, while carbon stored in DOM was projected to increase by 159.1 Tg C. For the period 2015–2100, the mean annual net flux from terrestrial ecosystems (−5.1 Tg C/year), was on average, 62% lower than the rate estimated for the historical period (−13.5 Tg C/year).

### 3.4 Effects of different scenario pathways

**Figure 5:**
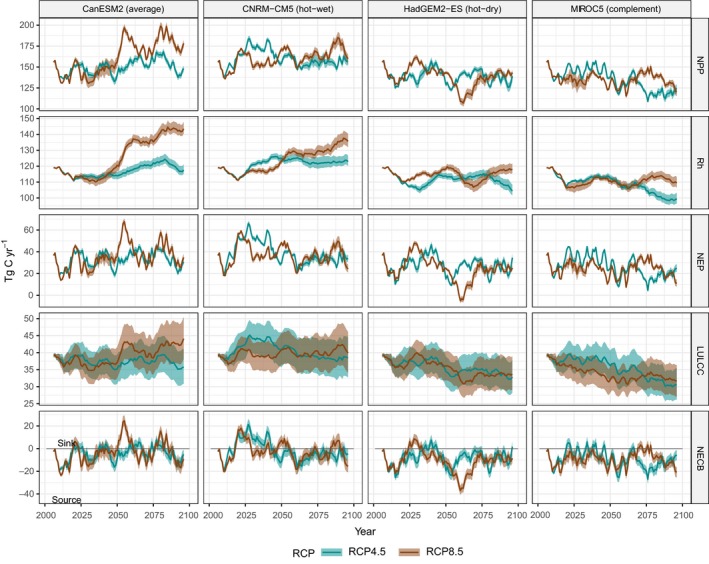
Net carbon fluxes, calculated as rolling 10 year averages, under the business‐as‐usual land‐use scenario for each of the eight climate pathways (representative concentration pathway [RCP] 8.5 shown in red, RCP 4.5 shown in green). Fluxes are shown from top to bottom as net primary productivity (NPP), heterotrophic respiration (Rh), net ecosystem productivity (NEP), losses from land use and land cover change (losses shown as positive values), and net ecosystem carbon balance (NECB). Rh includes carbon baseline emissions from litter and soil pools, NEP is estimated as NPP‐Rh, and NECB is estimated as NEP minus ecosystem carbon removals from land use, land‐use change, disturbance, and leaching. Negative values indicate a net loss of carbon from ecosystems

Climate models were the primary source of uncertainty in projected ecosystem carbon balance (Table 3; Figure 5). When averaged across all scenario simulations, mean annualized net carbon flux ranged from −8.4 to −0.8 Tg C/year. The largest cumulative declines in carbon storage were projected under the MIROC5 (‘Complement’; −713.5 Tg C) and HadGEM2‐ES (‘hot‐dry’; −703.7 Tg C) models. The smallest declines were associated with the ‘hot‐wet’ model (CNRM‐CM5) with a projected cumulative loss of −71.4 Tg C. The ‘averages’ model (CanESM2) was chosen specifically to represent an average of down‐scaled climate models for California (Bedsworth et al., 2017; Pierce et al., 2014) and projected a net decline of −240.6 Tg C by 2100 with a mean annualized rate of −2.8 Tg C/year. On average, simulations based on the RCP 8.5 pathway resulted in a loss of −481.8 Tg C from ecosystems, however, variability was large, with estimates ranging from a source of −925.8 Tg C/year to a net sink of 23.5 Tg C. Switching from the RCP 8.5 to RCP 4.5 climate pathway resulted in a reduction in net carbon losses of 20.5%. However, reductions in carbon losses were inconsistent, and highly dependent upon climate model. For example, when switching from the RCP 8.5 to RCP 4.5 pathway, ecosystems sequestered an additional 267.3 Tg C by 2100 under the ‘hot‐dry’ model (HadGEM2‐ES), while under the ‘average’ model (CanESM2), ecosystem carbon losses increased by 76.4 Tg C. Increases in precipitation under the CanESM2 RCP 8.5 scenario resulted in large increases in NPP which led to increased rates of sequestration not realized under the RCP 4.5 future (Table 3). These results suggest the overall benefits of global‐scale reductions in radiative forcing and reductions in atmospheric greenhouse gasses may not be realized at local‐to‐regional scales and may result in positive feedbacks on the climate system through reductions in ecosystem sequestration potential.

**Table 3:** Differences in mean annual projected carbon fluxes compared to historical period for each land use and RCP scenario as simulated by four climate models (GCM)

LUC	RCP	GCM	ΔNPP	ΔRh	ΔNEP	ΔLULCC	ΔNECB
BAU	RCP 4.5	CanESM2	4.2	−4.3	8.6	−1	9.6
		CNRM‐CM5	15	0.3	14.7	2.3	12.3
		HadGEM2‐ES	−6.8	−10.6	3.8	−3	6.8
		MIROC5	−11.9	−14	2.1	−3.2	5.3
	RCP 8.5	CanESM2	18.1	6.3	11.9	1.2	10.7
		CNRM‐CM5	16	3.4	12.6	1.4	11.2
		HadGEM2‐ES	−7.1	−7	−0.1	−3.4	3.3
		MIROC5	−12.2	−11.1	−1.1	−4.9	3.8
High	RCP 4.5	CanESM2	5.5	−4.7	10.3	0.9	9.3
		CNRM‐CM5	16.7	0	16.6	4.5	12.2
		HadGEM2‐ES	−5.6	−11	5.4	−1.1	6.5
		MIROC5	−10.8	−14.4	3.6	−1.5	5.1
	RCP 8.5	CanESM2	19.6	5.9	13.8	3.3	10.4
		CNRM‐CM5	17.5	3	14.5	3.6	10.9
		HadGEM2‐ES	−5.7	−7.3	1.6	−1.4	3.1
		MIROC5	−11.2	−11.5	0.3	−3.2	3.5
Medium	RCP 4.5	CanESM2	5.2	−3.7	9	−1.3	10.3
		CNRM‐CM5	16.5	1.2	15.3	2	13.3
		HadGEM2‐ES	−5.8	−10	4.2	−3.2	7.4
		MIROC5	−10.9	−13.4	2.5	−3.5	5.9
	RCP 8.5	CanESM2	19.6	7.2	12.4	0.9	11.5
		CNRM‐CM5	17.5	4.3	13.3	1.2	12
		HadGEM2‐ES	−6	−6.3	0.3	−3.5	3.8
		MIROC5	−11.4	−10.5	−0.9	−5.2	4.3
Low	RCP 4.5	CanESM2	7.1	−1.9	9.1	−2.3	11.3
		CNRM‐CM5	18.3	3	15.3	0.9	14.4
		HadGEM2‐ES	−4	−8.3	4.3	−4.1	8.5
		MIROC5	−9.3	−11.8	2.5	−4.5	7
	RCP 8.5	CanESM2	21.5	9	12.5	0	12.5
		CNRM‐CM5	19.2	6.1	13.2	0	13.1
		HadGEM2‐ES	−4.3	−4.5	0.3	−4.5	4.8
		MIROC5	−9.8	−8.8	−0.9	−6.1	5.3

Abbreviations: BAU, business‐as‐usual; GCM, global climate model; LUC, land‐use change; LULCC, land use and land cover change; NECB, net ecosystem carbon balance; NPP, net primary production; RCP, representative concentration pathway; Rh, heterotrophic respiration; TEC, total ecosystem carbon.

The four land‐use scenarios used in this study were developed to explore alternative future pathways based on low, medium, and high population and land‐use intensity assumptions, along with a scenario which explored a continuation of current trends in land use and LUC (e.g., BAU; Sleeter, Wilson, et al., 2017; Sleeter, Sleeter, et al., 2017). Projected changes in land use are shown in Figure S3. Urbanization was projected to occur primarily on agricultural lands, grasslands, and shrublands while changes in agriculture were primarily located in grassland and shrubland ecosystems. The ‘high’ scenario was the only scenario where agricultural lands were projected to increase in overall area (Figure S4). Under all scenarios, forest, grassland, and shrubland were projected to decline from current levels. When averaged across all climate model and RCP simulations, the ‘high’ scenario resulted in the largest net decline in ecosystem carbon balance (−521.7 Tg C) of the four land‐use scenarios (−6.1 Tg C/year). Under the ‘BAU’ scenario, which explored an extrapolation of recent historical rates of change, ecosystem carbon was projected to decline by a cumulative −479.2 Tg C by 2100 (−5.6 Tg C/year). Reducing the rate of various land use and LUCs (e.g., rate of timber harvest, urbanization, expansion of agriculture) consistently achieved reductions in ecosystem carbon losses, regardless of climate or RCP scenario. Relative to the ‘high’ land‐use scenario, ecosystems sequestered an additional −1.1 Tg C/year under the ‘medium’ land‐use scenario and −2.5 Tg C/year under the ‘low’ scenario.

The BAU and medium scenarios share the same set of assumptions, aside from the rate of urbanization. Under the BAU scenario, historical rates of urbanization were extrapolated into the future, while under the medium scenario, urbanization was based on a medium population growth projection (see Sleeter, Sleeter, et al., 2017; Sleeter, Wilson, et al., 2017 for additional details). When averaged across all GCM and RCP scenarios, California ecosystems sequestered an additional 57.4 Tg C when urbanization was reduced from the BAU (historical rates) to the medium scenario (e.g., slowing population growth after mid‐century). Under the ‘average’ climate model (CanESM2), urbanization reductions alone represented 17%–25% of NECB, while under the ‘hot‐wet’ model (CNRM‐CM5) and low emissions scenario (RCP 4.5), reducing urbanization accounted for 94% of NECB.

**Figure 6:**
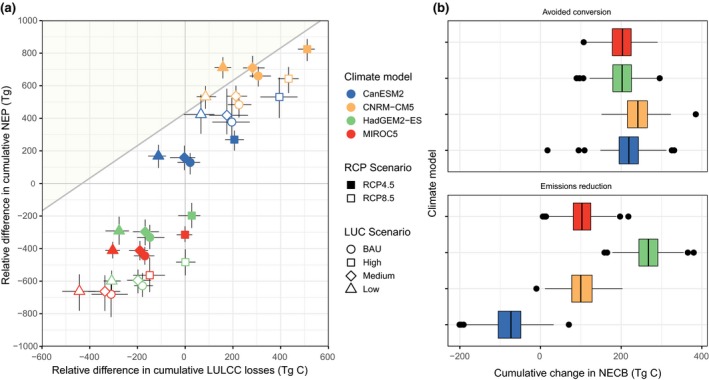
(a) Relative effects of climate change (*y*‐axis; increasing from top to bottom) and land use and disturbances (including wildfire; *x*‐axis, increasing from left to right) for each of the 32 scenario simulations for the period 2017–2100. Error bars represent the Monte Carlo confidence intervals calculated for each scenario. Shaded area represents the area within the plot space where ecosystems were estimated to be a net carbon sink over the projected period. All values are cumulative over the simulation period and were rescaled as the difference from the mean of the 32 scenarios. (b) Box‐plot showing the change in total ecosystem carbon for each climate model when switching from high to low land‐use scenarios (top; local land‐use mitigation achieved through avoided conversion) and switching from the representative concentration pathway (RCP) 8.5 to RCP 4.5 radiative forcing scenarios (bottom; global mitigation). Box‐plots show the median (black bar), 25th and 75th percentiles (boxes), 10th and 90th percentiles (whiskers), and outliers (points). Negative values indicate a net loss of ecosystem carbon storage while positive values indicate net carbon sequestration. LULCC, land use and land cover change; NECB, net ecosystem carbon balance

Figure 6 shows the cumulative estimate of NEP and NECB for each of the 32 scenarios used in this study. In general, the ‘hot‐dry’ (HadGEM2‐ES) and ‘complement’ (MIROC5) models, combined with high global emissions and high rates of LUC, resulted in the scenarios with the largest cumulative declines in ecosystem carbon (−916.2 and −846.9 Tg C by 2100, respectively). Under the ‘hot‐dry’ model, reducing global emissions resulted in similar rates of NPP and increases in NEP, indicating the avoided warming associated with the RCP 4.5 scenario decreases carbon losses associated with ecosystem respiration; NECB was projected to increase by 28.3%. Under the same ‘hot‐dry’ model, switching from the ‘high’ to the ‘low’ land‐use scenario resulted in a 21.1% increase in NECB while combining both global emissions reductions and reductions in land use resulted in net ecosystem carbon losses being reduced by 51.2%. Under the most optimistic scenario (low negative climate impacts, global emissions reductions, and low LUCs), California ecosystems were projected to be a net sink of carbon at a rate of 1.4 Tg C/year.

### 3.5 Effect of CO_2_ fertilization

**Figure 7:**
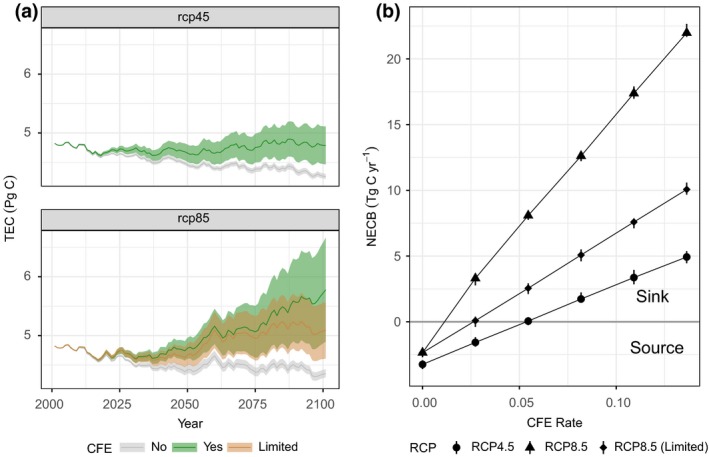
Effect of CO_2_ fertilization (CFE) on total ecosystem carbon storage (TEC) and net ecosystem carbon balance (NECB). (a) Projected TEC over time under three alternative CFE scenarios (No CFE, With CFE, and Limited CFE) for two climate models (representative concentration pathway [RCP] 4.5 and RCP 8.5). All CFE scenarios are based on the business‐as‐usual land‐use scenario and CanESM2 climate model. Colors show the projected mean, minimum and maximum estimates for scenarios with no CFE, with a range of CFE values, and with CFE limited to 600 ppm (under RCP 8.5 only). (b) CFE rate (percent change in NPP for a 100 ppm increase in atmospheric CO_2_) plotted against projected average NECB (from year 2001 to 2100). Points falling above the horizontal line at zero denote scenarios where California is a net sink of carbon while values below zero indicate a net source of carbon to the atmosphere

The effect of *C*_a_ enrichment on ecosystem productivity has a major impact on uncertainty in the estimation of regional scale carbon balance (Figure 7). The inclusion of a CFE effect with *β* values ranging from 0.027 to 0.136 resulted in increases in carbon sequestration by the end‐of‐century ranging from 1.7–12.4 Tg C/year (assuming the CFE effect was limited to *C*_a_ of 600 ppm). Assuming no CFE limitation based on *C*_a_, NECB was projected to increase between 5.7 and 24.3 Tg C/year under the RCP 8.5 scenario. Compared to the reference scenario (*β* = 0), total carbon stored in California ecosystems was projected to increase by 12.5% under RCP 4.5 and 17.1% under RCP 8.5 (limited at 600 ppm) assuming a moderate CFE (*β* = 0.082). For RCP 4.5, the moderate CFE effect was enough to offset all other carbon losses resulting in California ecosystems being relatively carbon neutral by the end of the century while under RCP 8.5, high rates of *C*_a_ accumulation result in rapid increases in carbon storage through the midcentury before leveling off after year 2060 when the CFE reaches saturation. Under the unlimited RCP 8.5 scenario, carbon sequestration continues unabated reaching a cumulative increase of 31.9% relative to the reference scenario.

## 4 DISCUSSION

Historically, California ecosystems have been a net source of carbon at an average rate of −13.5 Tg C/year. Estimates of the annual source–sink rate were high, and largely the result of interannual variability in weather and climate conditions and persistent extreme drought conditions. Major drivers of NECB included changes in vegetation productivity, ecosystem respiration, and the episodic nature of disturbances such as wildfire and drought. Future projections suggest ecosystems of California will continue to lose carbon at a mean annual rate of −5.1 Tg C/year. However, uncertainty in the source/sink rate was large, with estimates ranging from −10.8 to 1.4 Tg C/year. The large degree of uncertainty in the future source–sink rate was primarily due to differences in future climate conditions projected by climate models. Scenarios exploring the effect of global emission reductions (i.e., RCPs) did not achieve ecosystem carbon benefits across all climate futures, and may result in unanticipated positive feedbacks on climate forcing. Reducing land use consistently achieved reductions in net ecosystem carbon losses and was a reliable way of increasing carbon storage in ecosystems, regardless of future climate scenario. However, uncertainties resulting from the effect of increases in atmospheric CO_2_ are large, and must be reconciled in order to better constrain model projections.

### 4.1 Implications of different climate and land‐use pathways

By comparing various scenario combinations, we can determine the relative impact of both global adherence to a lower emission trajectory and jurisdictional actions to reduce emissions from land use and LUC (Figure 6). On average, the cumulative effect of a lower emission trajectory (i.e., from RCP 8.5 to RCP 4.5) changed net ecosystem carbon losses in California by −17% to 57% through 2100, with a mean estimate of 99 Tg C sequestered in terrestrial ecosystems. Global emission reduction had the largest positive impact under more extreme climate futures; for example, under the ‘hot‐dry’ future (HadGEM2‐ES), reducing global emissions leads to an additional 267.3 Tg C sequestered by ecosystems, resulting in a reduction of 31.9% in net ecosystem carbon losses. Conversely, under the ‘average’ climate future (CanESM2), the global emission reduction scenario resulted in a 37.7% increase in ecosystem carbon losses compared to the RCP 8.5 pathway, suggesting that local effects of reductions in global radiative forcing are uncertain and may result in a positive feedback to the climate system.

While the effect of global climate mitigation on ecosystem carbon balance in California can be variable, reducing LUC produced a reliable increase in carbon sequestration regardless of the climate future. Switching from the ‘high’ to the ‘low’ land‐use scenario resulted in a 37% to 46% reduction in net ecosystem carbon losses across all climate futures, with a median cumulative retention of an additional 215.3 Tg C in California ecosystems by 2100. The combination of global climate mitigation and a reduction in land‐use conversions results in the largest potential benefits to ecosystem carbon storage, reducing cumulative net losses by 316.1 Tg C. Under a ‘hot‐dry’ climate future, global emissions reductions combined with a shift from high to low rates of LUC reduced ecosystem carbon emissions by 51% (from −916.2 to −447 Tg C). Relative to global emission reduction scenarios, results suggest that reducing ecosystem carbon losses due to harvest and land use is an important and reliable approach for subnational jurisdictions like California to achieve greenhouse reduction goals and to reduce positive feedbacks to the climate system. These results highlight the benefits of subnational jurisdictions fully participating in reducing emissions from the energy and transportation sectors—thereby contributing to global mitigation—while reducing land‐use emissions to minimize potential climate‐driven ecosystem carbon losses.

### 4.2 Driving forces of carbon change

In this study, we estimated the direct effects of climate change on ecosystem carbon balance through both changes in vegetation productivity and the decay and decomposition of DOM. Additionally, projected changes in climate were used to estimate changes in the magnitude and frequency of natural disturbance events including wildfire and drought‐induced tree mortality. The climate models considered in this study provided a wide range of future conditions, with mean annual temperature increases ranging from 1.7 to 4.5 C by the end‐of‐century (Figure S5). The effect of increased warming resulted in large declines in soil carbon pools across almost all scenarios (Figure 4). Although more variable, changes in precipitation were important drivers of changes in NPP. For example, under the ‘average’ climate model (CanESM2) combined with the high emission scenario (RCP 8.5) and BAU land‐use scenario, NPP was projected to increase by 18.1 Tg C/year over the historical period (Table 3), owing primarily to hot‐wet conditions conducive to increased plant productivity. Conversely, under the same RCP and land‐use scenario, the ‘hot‐dry’ model (HadGEM2‐ES) projected declines in NPP of 7.1 Tg C/year relative to the historical period.

Projected emissions from wildfire under RCP 4.5 were consistent across GCMs and more pronounced under RCP 8.5. While all GCMs show an upward trend in projected wildfire emissions, the much higher emissions from CanESM2 were due to a steep increase in wildfire area during 2080–2100 (Figure S6). High‐ and medium‐severity wildfire also led to an additional pulse of carbon from live to DOM pools due to wildfire mortality. The effect of climate change on drought‐induced tree mortality was only evident in the high‐severity mortality class under RCP 8.5. High‐severity mortality led to a projected cumulative average transfer of 613 Tg C from the live to the DOM pools. In all scenarios, extreme episodic mortality events were driving carbon losses rather than consistent low‐level background events (Figure S6). This is characteristic of severe drought periods driven by a combination of low precipitation and high temperatures, or high temperatures alone. Under RCP 4.5, high‐severity mortality led to average cumulative losses of 563 Tg C from the live biomass pool, but no GCM differed more than 6% from this mean value.

### 4.3 Comparison to other studies in California

The comprehensive nature of this study, which used a gain–loss method to estimate carbon stocks and fluxes for California's forest, grassland, shrubland, and agricultural ecosystems over both recent historical conditions as well as 32 alternative future pathways, represents a unique contribution to understanding the interactions and major controlling processes of ecosystem carbon balance. As such, we can compare our estimates of carbon stocks and fluxes with other recent studies which have been more limited in scope, often covering only a subset of the variables included in this study.

A recent study (Gonzalez, Battles, Collins, Robards, & Saah, 2015) estimated that in 2001, California ecosystems stored 920 Tg C in live aboveground vegetation, of which 830 Tg C was stored on 125,000 km^2^ of land classified as forest. Our study estimated California forests stored 1638.8 Tg C in total live biomass carbon (sum of above‐ and belowground stocks). While we did not explicitly model above and below ground carbon stocks as separate pools, we can infer the aboveground portion by applying a standard root:shoot relationship of 0.27 (Mokany, Raison, & Prokushkin, 2006). This provides an estimate of forest live aboveground carbon storage of 1,196.3 Tg C in 2001. Comparisons are further complicated due to differences in land classification, particularly differences in the extent and amount of forest area. We estimated the 2001 forested land area in California was ~156,000 km^2^, which was 20% higher than the estimate by Gonzalez et al. (2015). Normalizing for differences in the extent of forest area provides an estimate of 957 Tg C stored in forest aboveground live stocks, an amount more comparable to that provided by Gonzalez et al. (2015) (830 Tg C). For forests, the stock change study (Gonzalez et al., 2015) estimated a decline of −50 Tg C (−5.6 Tg C/year) between 2001 and 2010 which was approximately two times larger than our estimate of −25.6 Tg C (−2.8 Tg C/year) over the same period. However, the stock change approach did not include carbon accumulation (e.g., growth) on forest cells which did not change from one ordinal forest class (e.g., forest type, cover, and height) to another (Gonzalez et al., 2015), whereas this study's gain–loss approach estimated growth on an annual basis for all simulation cells. A comparison of this study to other published research estimating ecosystem carbon stocks in California is shown in Table S1.

Our estimates of statewide NPP are within 15%–25% of those from satellite‐derived estimates (Zhao, Heinsch, Nemani, & Running, 2005) for the historical period (2002–2015), but are consistently lower and have higher year‐to‐year variability (Figure S8), suggesting the LUCAS model estimates are more sensitive to interannual climatic variability. Spatial and temporal variability in LUCAS‐estimated NPP is represented by an empirical NPP model (Del Grosso et al., 2008) which was calibrated using NPP estimates from the Ecosystem Model‐Data Intercomparison (EMDI) project which consists, in part, of thousands of regional‐scale NPP estimates based on growth increment from the Forest Inventory and Analysis database (Olson, Scurlock, Prince, Zheng, & Johnson, 2013). Satellite‐derived estimates were based on the MOD17 algorithm incorporating spectral reflectances and meteorological scalars (Zhao, Running, Heinsch, & Nemani, n.d.), which tends to overestimate NPP in low productivity ecosystems (Turner et al., 2006) that dominate much of California. In addition, MOD17‐based estimates of productivity tend to be insensitive to interannual variation and drought effects in ecosystems dominated by evergreen vegetation, which make up the majority of California's forests and shrublands (He et al., 2016; Hwang et al., 2008; Porcar‐Castell et al., 2014; Verma et al., 2015).

Wildfire is a major source of carbon emissions to the atmosphere in California. The California Air Resources Board (ARB) estimates carbon emissions from wildfire (California Air Resources Board, 2019) using the First Order Fire Effects Model (FOFEM) developed by the US Forest Service (Reinhardt, Keane, & Brown, 1997) with fire footprints from CalFIRE (2016). Estimates from our study compare well with those from ARB over the overlapping historical period (2002–2016) and are shown in Figure S9. We estimate that historical fire emissions averaged 14.3 (*σ* = 12.6) CO_2_ per year compared to an average historical rate of 15.5 (*σ* = 11.2) CO_2_ per year based on the ARB methodology. Annual estimates and variability also show a strong agreement with estimates produced using the ARB modeling approach with an *r* ^2^ value of 0.92 based on a comparison of annual emission estimates.

### 4.4 Limitations of study

This study was limited to just 4 of the 32 LOCA down‐scaled global climate models from the CMIP5 archive. While these four models were chosen by a working group for the California 4th Climate Assessment as representing a broad range of future California climates, our results would likely be different if all 32 models were assessed in this framework. More importantly, our current framework did not include variability in key parameters that would likely increase the uncertainty of the results. This includes the uncertainty associated with parameters related to climate (i.e., effect of precipitation and temperature on NPP and DOM turnover rates), carbon (base stocks and flows), and disturbance (extent of wildfire and mortality). Our approach did not account for changes in vegetation type which may result from the coupled effects of climate change and high‐severity fire. Specifically, there is growing concern that in some regions, the ability of forests to recover after large high‐severity fires is reduced in response to climate change (Thorne et al., 2017). Our model did not estimate these effects and may result in an overestimation of carbon storage given the assumption that forests will always recover after disturbance.

Future warming, and its effect on DOM turnover rates, was represented using climate model temperature projections and a Q10 function generally consistent with those used in the Carbon Budget Model of the Canadian Forest Sector (CBM‐CFS3; Kurz et al., 2009). However, the CBM‐CFS3 model does not include a Q10 for the decomposition of the slow recalcitrant pool, which might indicate our model overestimates the temperature sensitivity of decay rates of SOC. However, a recent whole‐profile warming experiment in California (Pries et al., 2017) determined an effective Q10 for soil CO_2_ efflux to be 2.4, suggesting our estimate of SOC temperature sensitivity may be conservative.

While rising atmospheric CO_2_ undoubtedly plays a role in vegetation productivity, the magnitude and duration of this effect and how it manifests in specific ecosystems is highly uncertain (Smith et al., 2016), especially under long‐term scenario projections (Arora et al., 2013; Friedlingstein et al., 2014; Piao et al., 2013; Sitch et al., 2008). Early results from FACE studies appeared to support this strong positive CFE on NPP at the ecosystem level across a range of forest types (Norby et al., 2005). However, there is also substantial evidence that an array of feedback responses and constraints can dampen or eliminate CO_2_‐induced increases in plant carbon assimilation and growth (Franks et al., 2013; Norby et al., 2010; Smith & Dukes, 2013). Longer term results from FACE studies in both forests and grasslands have demonstrated that the initial CFE‐induced growth stimulation declined over time as nitrogen (N) became more limiting (Norby et al., 2010; Reich et al., 2006). The magnitude of this progressive N limitation on CFE is highly site‐specific, and sometimes does not appear to dampen CFE at all because of high site N status or positive feedbacks to N cycling (Smith & Dukes, 2013). Moreover, FACE experiments represent a step change in *C*_a_ of often ~150%. Results from these experiments can be difficult to interpret in the context of gradual, year‐by‐year increases in *C*_a_ over decades, especially at regional to continental scales. Studies based on remote sensing have also not been able to constrain the CFE effect on NPP. For example, although CFE explains 70% of increased plant greenness over time at a global scale (Zhu et al., 2016), a comparison with ecosystem models demonstrated that models overestimate CFE‐induced increases in NPP by ~150% compared to satellite‐based estimates over a 30‐year period (Smith et al., 2016).

The *β* values included in the sensitivity analysis generally cover the range of values observed at forested FACE sites (Norby et al., 2005; Smith et al., 2016; Zaehle et al., 2014). These estimates tend to be higher than *β* values derived from a remote sensing–based approach (Smith et al., 2016), which have been criticized as likely underestimating the CFE (De Kauwe, Keenan, Medlyn, Prentice, & Terrer, 2016), and considerably lower than values reported in a range of Earth System Models (see figure 4 in Smith et al., 2016), indicating that the uncertainty in ecosystem carbon storage (TEC) and flux (NECB) is likely to be much larger than the estimates shown in Figure 7. The large degree of uncertainty resulting from the introduction of a range of CFE's in this study underscores the need for more research on the role of increasing atmospheric CO_2_ on terrestrial carbon cycling, especially under long‐term projections, as studies have indicated that the role of increasing CO_2_ on ecosystem carbon balance can be as much as four times larger than the effect of climate change (Arora et al., 2013; Gregory, Jones, Cadule, & Friedlingstein, 2009). In the context of climate mitigation planning, assuming no CFE is the most conservative approach and that any realized CFE in the future will likely only further increase carbon sequestration in ecosystems.

Similar to most other carbon cycle models, our model might overestimate recovery rates of carbon sink strength after prolonged drought (Anderegg et al., 2015). Between 2013 and 2015, California entered a period of sustained and exceptional drought which affected most areas and ecosystems of the State. Evidence suggests the extreme drought conditions experienced in recent years were without precedent over instrumented and millennial records (Robeson, 2015), and while primarily the result of natural climate variability (Berg & Hall, 2015; Williams et al., 2015), anthropogenic warming was an import contributing factor (Diffenbaugh et al., 2015; Shukla, Safeeq, AghaKouchak, Guan, & Funk, 2015; Williams et al., 2015). In this study, the effect of drought conditions on ecosystem carbon balance was significant. Between 2013 and 2015, NECB was estimated at −52.3 Tg C/year with an estimated loss of −90 Tg C/year in 2013 alone, and was largely driven by a large negative precipitation anomaly (Figure S7). The range of climate models and RCP scenarios used in this study produced a single time step with a 1 year decline in NECB similar to that of 2013 (−98.8 Tg C/year; CanESM2, RCP 8.5 in 2096). Under the RCP 8.5 scenario, three of the four climate models (CanESM2 in 2063, HadGEM2‐ES in 2058–2059, and MIROC5 in 2059) projected NECB declines similar to that of the recent drought period, suggesting that while the probability of a single year event such as 2013 was low, severe multiyear drought conditions in the future are represented in future projections. Of particular note is the projection of a sustained drought period occurring midcentury under the RCP 8.5 scenario as simulated by the HadGEM2‐ES climate model; between 2051 and 2070, ecosystems were estimated to be a net source of carbon in 16 of 20 years (−22.9 Tg C/year), a rate nearly twice as high as the 14 year historical average. To better understand the context of these extreme events, future work should extend the baseline period of analysis to encompass additional periods of severe drought in California (e.g., 1976–1977, 1987–1992). While the uncertainty associated with projecting extreme events is large (Swain et al., 2014), substantial evidence suggests anthropogenic climate change will increase the probability of extreme drought conditions in California (Cook et al., 2015; Diffenbaugh et al., 2015; Williams et al., 2015) which may have significant impacts on ecosystem carbon balance.

## 5 CONCLUDING REMARKS

Changes in ecosystem carbon balance are an important driver of global climate change. However, there is a great deal of uncertainty in the direction and magnitude of the carbon source or sink. Results of this study show that uncertainty primarily resulted from future climate conditions driven by different climate models and are consistent with other studies in this regard (Zaehle et al., 2007). However, these uncertainties are relatively small compared to the uncertainties associated with increasing CO_2_ and its effect on carbon storage and flux. While NECB was projected to increase relative to recent historical rates, carbon stored in California ecosystems was projected to decline in 30 of the 32 future scenarios considered in this study. Reducing global greenhouse gas emissions did not always result in increased carbon storage in California ecosystems, suggesting there may be unanticipated feedbacks to the climate system resulting from ecosystem carbon flux. Conversely, reducing LUC was a reliable and consistent approach to increasing carbon sequestration, regardless of future climate conditions. These findings are useful for establishing a set of baseline projections from which additional ecosystem‐based climate mitigation strategies can be evaluated (Cameron et al., 2017; Fargione et al., 2018). Such studies are needed to understand the role of ecosystems in regulating greenhouse gas emissions and for achieving local to global‐scale climate mitigation goals.

## CONFLICT OF INTEREST

The authors declare no competing interests.

## AUTHOR CONTRIBUTIONS

BMS was the lead researcher, developed and parameterized the model, analyzed the results, and drafted the manuscript. DCM helped in all aspects of the research including model development and parameterization, data analysis, and writing of the manuscript. DRC assisted in model development, analysis, and writing. PCS assisted in analysis and writing. LW developed wildfire projections. CJD contributed to model development and writing, JL developed carbon flux parameters. TSW contributed to writing. DCM and JK implemented the model on a high‐performance computing environment.

## CODE AND DATA AVAILABILITY

The model, source code, and data required to replicate this study, as well as the output data supporting the conclusions of the study are available through the USGS ScienceBase repository.

The LUCAS model runs within the Syncro‐Sim software application. All simulations were run using Syncro‐Sim software version 2.0.18, under the Mono framework for Linux. The STSM and SF modules used in this study were version 3.1.18. We ran the simulations on the Comet system at the San Diego Supercomputing Center under the NSF Extreme Science and Engineering Discovery Environment (XSEDE) program through allocation TG‐DEB17001767.

The following steps are required to run the model used in this analysis:

- Download and install the latest Windows or LINUX version of the Syncro‐Sim software, available at http://www.apexrms.com.
- Download and unzip the model files, including spatial input files and a ‘.ssim’ (SQLite) database from the online repository (to be filled in upon publication).
- Use the Syncro‐Sim software to open the ‘California Carbon Model.ssim’ file and select a scenario to run.
- Alternatively, the model can be built from scratch using the R programming language with the rsyncrosim package installed. To follow this approach, download the ‘California Carbon Model R Code.zip’ data package and run the necessary R scripts.

All output results from the 32 scenarios described in this report are available from the ScienceBase online repository. Each scenario includes an SQLite database (SyncroSim ‘.ssim’ file) and a compressed folder with all spatial output. Tabular output are contained entirely within each SQLite database. Each database file is ~36 GB and the corresponding compressed spatial output maps are an additional ~18 GB. The entire library is ~1.7 Tb. Given the large volume of data, several intermediate products were generated from which all results present in the paper were derived.

This paper was compiled using R Markdown. All results, including tables, figures, and values presented within text can be auto‐generated by downloading and running the R Markdown file including in the data repository. The data used within the R Markdown file is dependent upon a number of data summaries which were generated and written to disk and are stored in the ‘Data>Report_Tables’ folder.

Tabular data summaries are archived here: https://doi.org/10.5066/P9KVF795

Model and code are available on Github: https://github.com/bsleeter/california-carbon-scenarios
