# Supplementary material for: Effects of 21st‐century climate, land use, and disturbances on ecosystem carbon balance in California
Source: Glob Chang Biol. 2019 Jun 24;25(10):3334–53. doi: 10.1111/gcb.14677 (PMC6851753; doi:10.1111/gcb.14677)
Supplement: Supplementary file 1 [file GCB-25-3334-s001.pdf]

Effects of 21st century climate, land use, and disturbances on ecosystem carbon balance in California

Supplemental Material

Running Title: Ecosystem carbon balance in California

Benjamin M. Sleeter[1]*, David C. Marvin[2], D. Richard Cameron[2], Paul C. Selmants[3], LeRoy Westerling[4], Jason Kreitler[5], Colin J. Daniel[6], Jinxun Liu[3], Tamara S. Wilson[3]

Affiliations

[1]U.S. Geological Survey, Seattle, WA, USA; bsleeter@usgs.gov; (253) 343-3363

[2]The Nature Conservancy, San Francisco, CA, USA

[3]U.S. Geological Survey, Menlo Park, CA, USA

[4]University of California Merced, California, USA

[5]U.S. Geological Survey, Boise, ID, USA

[6]Apex Resource Management Solutions Ltd., Ottawa, Alberta, CAN

Keywords: land use, climate change, carbon balance, California, scenarios, disturbance

Date: March 13, 2019

Supplemental Methods

Wildfire submodel

For each timestep between 2001 and 2016, wildfire was estimated deterministically using annual burn perimeters from a state fire database (FRAP) (CalFIRE, 2016). Over the historical period, fire severity was modeled based on the relative proportion of each severity class (low, medium, and high) calculated from an analysis of annual burn severity maps (1985-2014) from the Monitoring Trends in Burn Severity (MTBS) database (Eidenshink et al., 2007). Between 2017-2100, burn area was estimated for each climate model (GCM) and radiative forcing scenario (RCP) based on a statistical model of wildfire which considered the effects of climate, vegetation, population density, and fire history (Westerling, 2018). The exogenous statistical fire model was used to derive burn area projections for each climate model, radiative forcing scenario, and land-use scenario (Sleeter, Wilson, Sharygin, & Sherba, 2017). For each scenario, 100 stochastic simulations were run and summarized to produce a time series of maps where each 1/16 degree cell had a projected mean burned area. The spatial maps were then summarized to provide a mean and standard deviation of total burn area for each ecoregion considered in this study.

The LUCAS model simulated individual fire events which spread across the landscape within a timestep. Fire events are projected based on 1) the expected annual burn area within an ecoregion, 2) the relative probability of an individual cell experiencing a fire, 3) the distribution of fire size, and 4) the distribution of fire severity classes. Within each ecoregion, the annual burn area was sampled, with replacement, from the distribution of burn area based on the statistical fire model. To preserve the spatial pattern of fire projected by the statistical model, we calculated the relative probability of fire for each 1/16 degree cell based on the mean estimated burn area in each timestep. Both fire size and severity were assumed to be stationary and were sampled based on historical data from FRAP and MTBS, respectively. Carbon fluxes associated with fire were based on Sleeter et al. (2018).

Drought-induced tree mortality submodel

To estimate the effects of climate on forest carbon we developed a model of drought-induced tree mortality. We used annual forest mortality data coinciding with two multi-year extreme drought periods that peaked in 2004 and 2016 and resulted in widespread tree mortality, especially for the latter drought period (Stephens et al., 2018). We used the US Forest Service Aerial Detection Survey (Moore, McAfee, & Iaccarino, 2018) annual tree mortality data, partitioned into low, medium, and high tree mortality classes (1-10, 11-20, and >20 trees per acre, respectively). We used the 60-month Standardized Precipitation Evapotranspiration Index (SPEI) (Vicente-Serrano, Beguera, & Lopez-Moreno, 2010) to track long-term drought annually across California using PRISM (PRISM Climate Group, Oregon State University, 2016) 4-km historical climate data for monthly temperature and precipitation inputs, using the Thornwaite method to calculate potential evapotranspiration. We fit a binomial GLM model for each of the three mortality classes, using SPEI as a single predictor in the model. We used these models to spatially predict future drought-induced mortality for each climate model and radiative forcing scenario on an annual timestep. We estimated the annual mortality area for each ecoregion from the model outputs and sampled, with replacement, from a Gaussian distribution created from these annual ecoregional means and an assumed 50% standard deviation. We constructed annual relative probability maps from the spatial predictions and used this to constrain the pattern of disturbance. See SI Methods for more detail. We sampled from a uniform distribution using proportional carbon flux ranges of 0.01-0.10, 0.10-0.5, and 0.5-1.0 for the low, medium, and high tree mortality classes, respectively.

Soil Carbon

We calculated soil carbon stock at standard intervals using soil organic carbon and bulk density produced for the contiguous U.S. at 100 m spatial resolution (Hengl et al., 2017, 2014; Ramcharan et al., 2018), and coarse fragments (>2mm) produced globally at 250 m spatial resolution (Hengl et al., 2017). We summed the carbon stocks over the depth intervals

from 0-100 cm, and re-sampled to 1-km using mean re-sampling. SOC estimates from the SoilGrids 250-m global product explained 69% of the variation in observed data based on 10-fold repeated cross-validation (Hengl et al., 2017). A separate comparison of multiple SOC estimates from global databases suggested the SoilGrids data product yielded the most accurate results at both global and regional scales (Tifafi, Guenet, & Hatté, 2018).

Effects of climate variability and change on net primary production (NPP)

Annual variation in growth was estimated based on an empirical model of NPP (Del Grosso et al., 2008) and annual climate model projections of mean annual temperature and total precipitation (Pierce, Cayan, & Thrasher, 2014) and is described in detail in Sleeter et al. (2018). The NPP model is based on an empirical relationship between total (above- and below-ground) NPP and mean annual precipitation (MAP) for non-tree dominated ecosystems (shrublands and grasslands); for forest ecosystems the equation includes both MAP and mean annual temperature (MAT) as predictor variables. Parameters in these equations were optimized by minimizing root mean square error (RMSE) for modeled and observed TNPP, which ensures that the mean predicted TNPP value will be nearly identical to the mean observed value. Regional model estimates of forest TNPP compare well with those derived from satellite data (1% difference) and biogeochemical process models (12% difference) (Cleveland et al., 2015). A spatially explicit stationary growth multiplier was used to scale the growth on individual cells to reflect variations in productivity due to local environmental site conditions. The spatial growth multiplier was estimated by calculating the NPP anomaly for each simulation cell relative to its ecoregional mean based on 30-year climate normals (PRISM Climate Group, Oregon State University, 2016).

We chose not to incorporate a CO_2 fertilization effect (CFE) on NPP into our scenarios. Although many biochemical reaction rates increase in response to increased substrate concentration, there is growing evidence that other factors may limit the effect of rising atmospheric CO_2 on net carbon assimilation by plants. Satellite-derived estimates of NPP suggest that

Earth system models overestimated the CFE by 50% over a 30-year period (Smith et al., 2016), and data from free air carbon dioxide enrichment (FACE) studies indicate the CFE was reduced or disappeared entirely under limitation by water and nutrients (Reich, Hobbie, & Lee, 2014) or extreme weather conditions (hotter, drier, or wetter) (Obermeier et al., 2017). The magnitude and persistence of a CFE on NPP under future climates is unresolved, so we were unable to parameterize a CFE effect based on available data.

Effects of climate warming on heterotrophic respiration (Rh)

Future warming, and its effect on DOM turnover rates, was represented using climate model temperature projections and a Q10 function. We assumed a Q10 of 2.0 for the decay of down deadwood, decomposition of litter to the soil pool, and gaseous emissions from the soil pool, and a Q10 of 2.65 was assumed for gaseous emissions from the litter pool. These rates are generally consistent with those used in the Carbon Budget Model of the Canadian Forest Sector (CBM-CFS3) (Kurz et al., 2009). The CBM-CFS3 model does not include a Q10 for the decomposition of the slow recalcitrant pool, which might indicate our model overestimates the temperature sensitivity of SOC decay rates. However, a recent whole-profile warming experiment in California determined an effective Q10 for soil CO_2 efflux to be 2.4 (Pries, Castanha, Porras, & Torn, 2017), suggesting our estimate of SOC temperature sensitivity may be conservative. Similar to the approach used to estimate temporal and spatial variability in NPP, a stationary spatial multiplier was used to reflect within ecoregion variability in DOM/SOC turnover based on 30-year climate normals. Next, for each GCM and RCP, ecoregion scale non-stationary temporal multipliers were used to reflect changes based on projected temperature.

Perennial croplands and age

We created a custom classification of the location and age of orchard croplands across California using a machine learning algorithm and a stack of satellite images and derivative

products. Our training/testing data-set consisted of field-level vector data of crop types obtained from agricultural commissioners from seven broadly representative agricultural counties. To assess orchard age, we used spectral unmixing to create an annual time series of bare ground fractional cover and created a metric to identify the occurrence of new orchard establishment that accounts for background variability in bare ground exposure of agricultural fields.

We created a custom classification of the location and age of perennial croplands across California because of a lack of perennial crop separation from other agricultural types (i.e. NLCD) (Homer et al., 2015) or the low local accuracy from data-sets like the US Cropland Data Layer (CDL) (Boryan, Yang, Mueller, & Craig, 2011). Our evaluation of CDL orchards against a field-level California-specific data layer commissioned by the Department of Water Resources (DWR) (California Department of Water Resources, 2017) with 97.4% orchard accuracy, found a statewide accuracy of only 64% for CDL for 2014. We excluded vineyards because of their low above-ground biomass relative to orchards. Our training and testing data-set consisted of county agricultural data from seven broadly representative counties (Butte, Colusa, Fresno, Merced, Monterey, Sonoma, Yolo) using field-level geospatial data from 2010-2011. The final data-set consisted of 10,000 randomly sampled points for orchards and 90,000 randomly sampled points for non-orchards (evenly split among other agriculture classes and natural vegetation). We used predictors composed of Landsat 5 surface reflectance bands for three different seasons in California (December-March, April-August, September-November) broadly corresponding to vegetation responses to precipitation. In addition, we included the NIRv vegetation index (Badgley, Field, & Berry, 2017) for each season, fractional land cover using spectral unmixing (shade, bare ground, vegetation, and urban) derived from the Landsat Greenest Pixel data product (Chander, Markham, & Helder, 2009), elevation (Gesch et al., 2002), and slope as predictors. All data were obtained and pre-processed using Google Earth Engine, and re-sampled to 100 meter resolution. We trained a model for 2010 using a gradient boosting machine (GBM) algorithm (Candel, Parmar,

LeDell, & Arora, 2016) with 10-fold cross validation and an exhaustive hyperparameter search. The 2010 model had a final validation (using a 10% holdout from the training data) accuracy of 86.8% and reliability of 91.9%. We then applied this model to prediction data from 2001 in order to generate a map of predicted orchards in California for that year. We assume this model is generalizable to previous years as all predictors are derived from the same satellite sensor (Landsat 5), with the exception of the elevation and slope, which are not expected to have changed. There is no available validation data from 2001 to create a statewide assessment for this layer. The map was re-sampled to 1-km using mode re-sampling.

Existing data layers also lacked orchard age, which is needed to produce refined estimates of orchard carbon stocks. To assess orchard age, we created an annual (1985-2001) fractional land cover using spectral unmixing (shade, bare ground, vegetation, and urban) derived from the Landsat Greenest Pixel data product (Chander et al., 2009). For every pixel identified as orchard, we used the bare ground fractional cover layer to find the year where the coefficient of variation across the entire time period crossed below a threshold of 1. We found this metric indicative of one or two years post-orchard removal after extensive manual testing using NAIP imagery as the ground truth. This 30 meter resolution pixel-level age map was passed through a majority filter with a kernel size of 150 meters (close to the minimum field size of 2.25 ha). This smoothed age map was re-sampled to 1-km using mode re-sampling.

Forest Age

We created a forest age map for the year 2001 using a combination of the Gradient Nearest Neighbor Forest Structure Stand Age (GNN Age) (Landscape Ecology, Modeling, Mapping, and Analysis (LEMMA), 2018; Ohmann, Gregory, Henderson, & Roberts, 2011), Monitoring Trends in Burn Severity (MTBS) (Eidenshink et al., 2007), and North American Forest Dynamics (NAFD) (Goward et al., 2012). We clipped all layers to California and re-projected them to the same extent and pixel dimensions. We extracted the high burn severity class from the 1984-2001 MTBS layers, assuming this to be a stand-age resetting event. We converted

all the high burn pixels to age since fire using 2001 as the anchor point, and combined them into a single layer by taking the minimum value across all layers. We used the 'last disturbance year' NAFD data layer and the GNN stand age layer and converted both to year since disturbance using 2001 as the anchor point. We combined all three of these into a single stand age at 2001 layer taking the minimum value.

Supplemental Figures

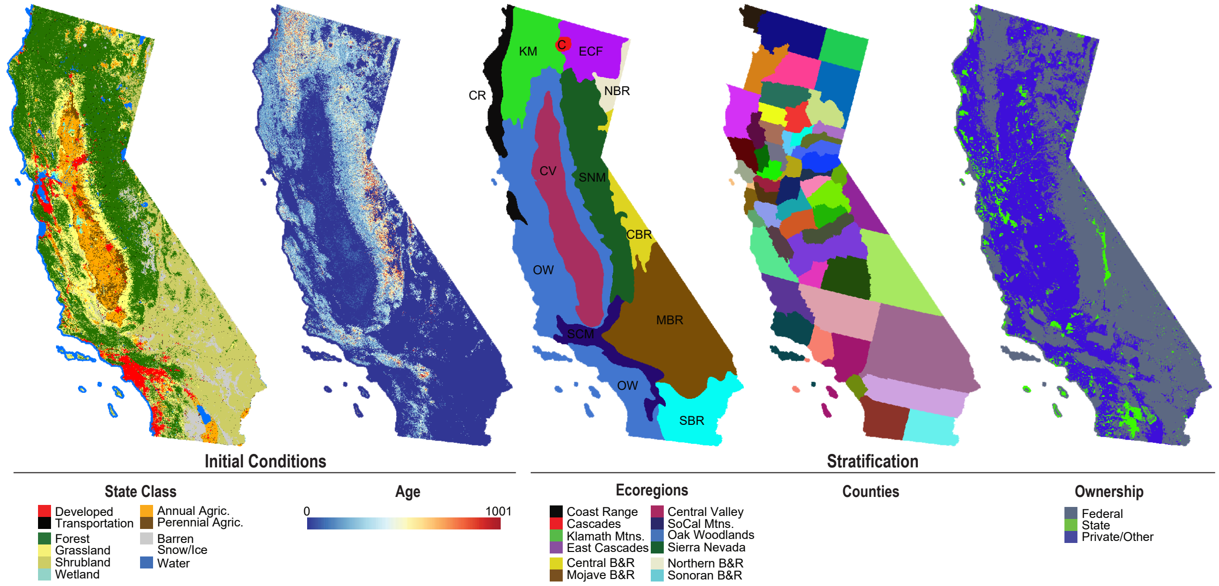

Figure 1: Maps of initial conditions and strata used in the LUCAS model. State class type was estimated based methods described in Sleeter et al., 2017. Forest age was estimated based on a gradiant nearest neighbor approach and described in the methods. Ecoregions were based on the U.S. EPA's Level III classification. County boundaries were derived from the U.S. Census Bureau's TIGER boundary files. Ownership was derived from the U.S. Geological Survey's Protected Areas Database

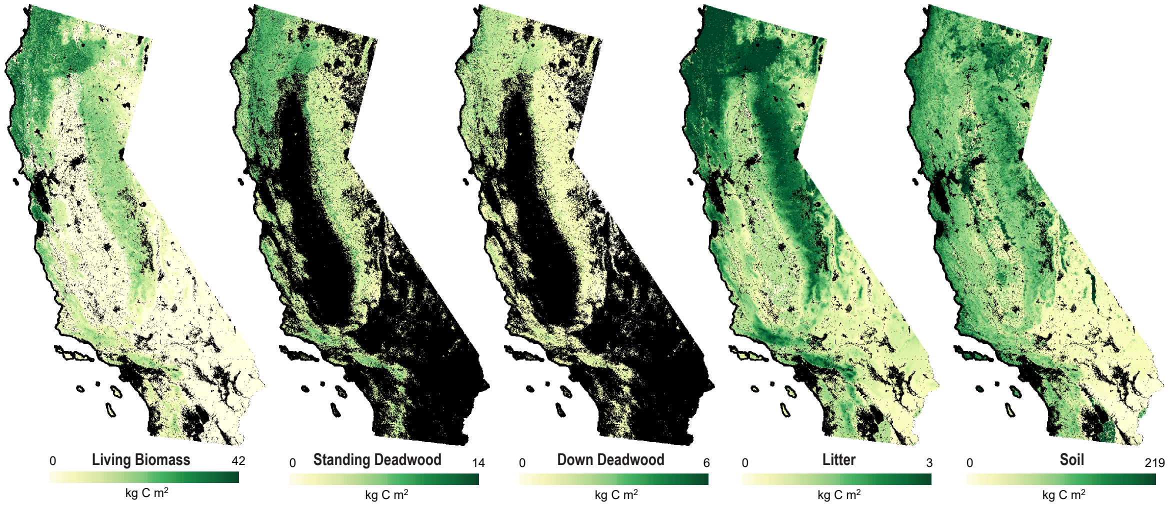

Figure 2: Maps of model initial carbon stocks. Initial stocks were estimated based on the ecoregion, state class type, and age of each simulation cell using a look-up table derived from a dynamic global vegetation model (DGVM). Values were further scaled based on a spatialy explicit growth multiplier calculated using 30-year climate normals and an empirical model of NPP. See the materials and methods section for additional details, as well as Daniel et al., 2018.

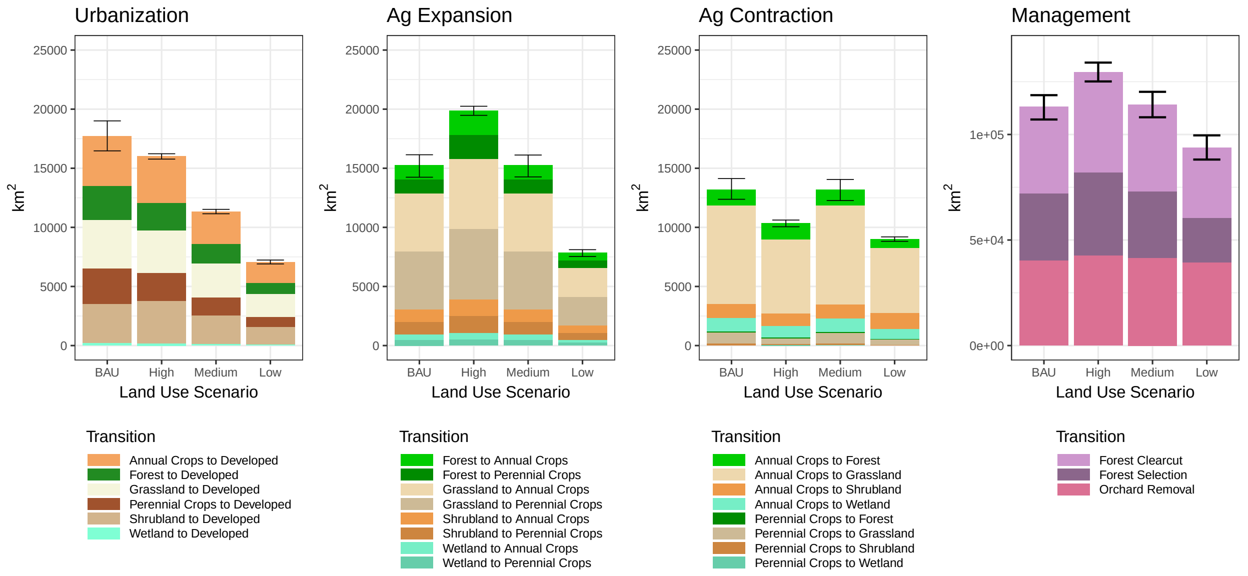

Figure 3: Mean cumulative (2001-2100) transition area for urbanization, agricultural expansion, agricultural contraction, and management transitions considered in this study. Bars show the mean estimated area for each land-use scenario averaged over all model simulations. Colored bar components show the specific from-to transition associated with each conversion type. Error bars show the Monte Carlo confidence intervals for the total cumulative conversion area.

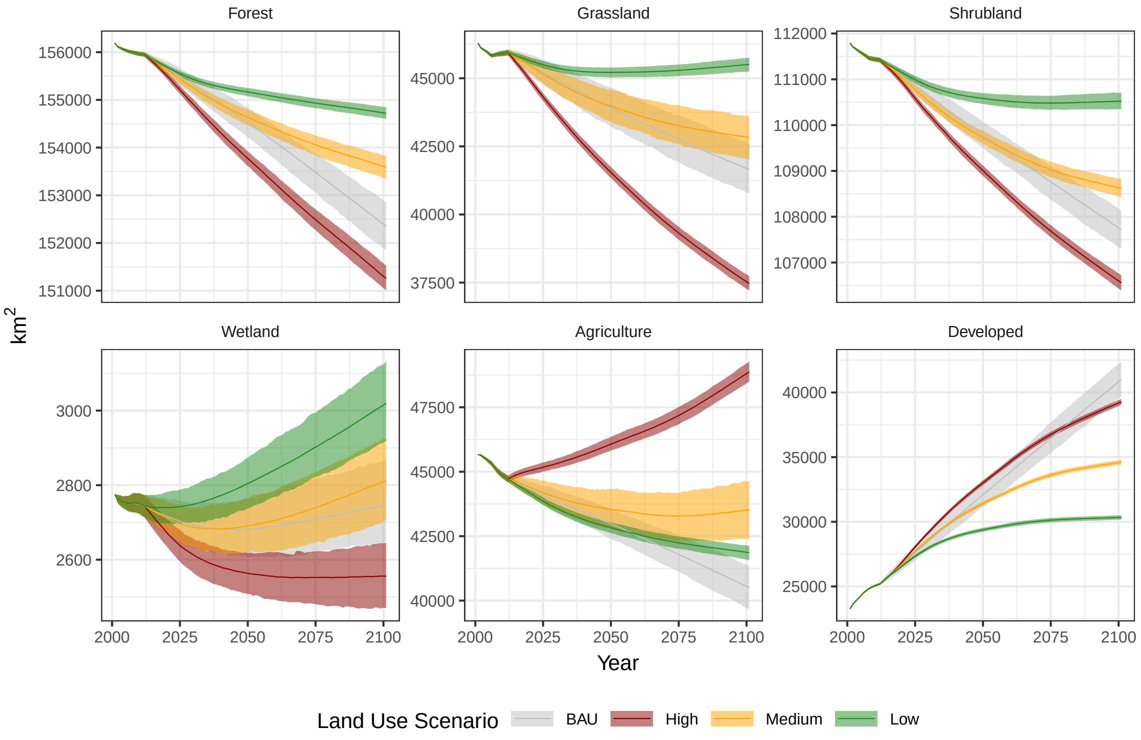

Figure 4: Estimated state class area for each land-use scenario. Lines show the mean estimate and ribbons show the Monte Carlo confidence intervals calculated over all scenario simulations.

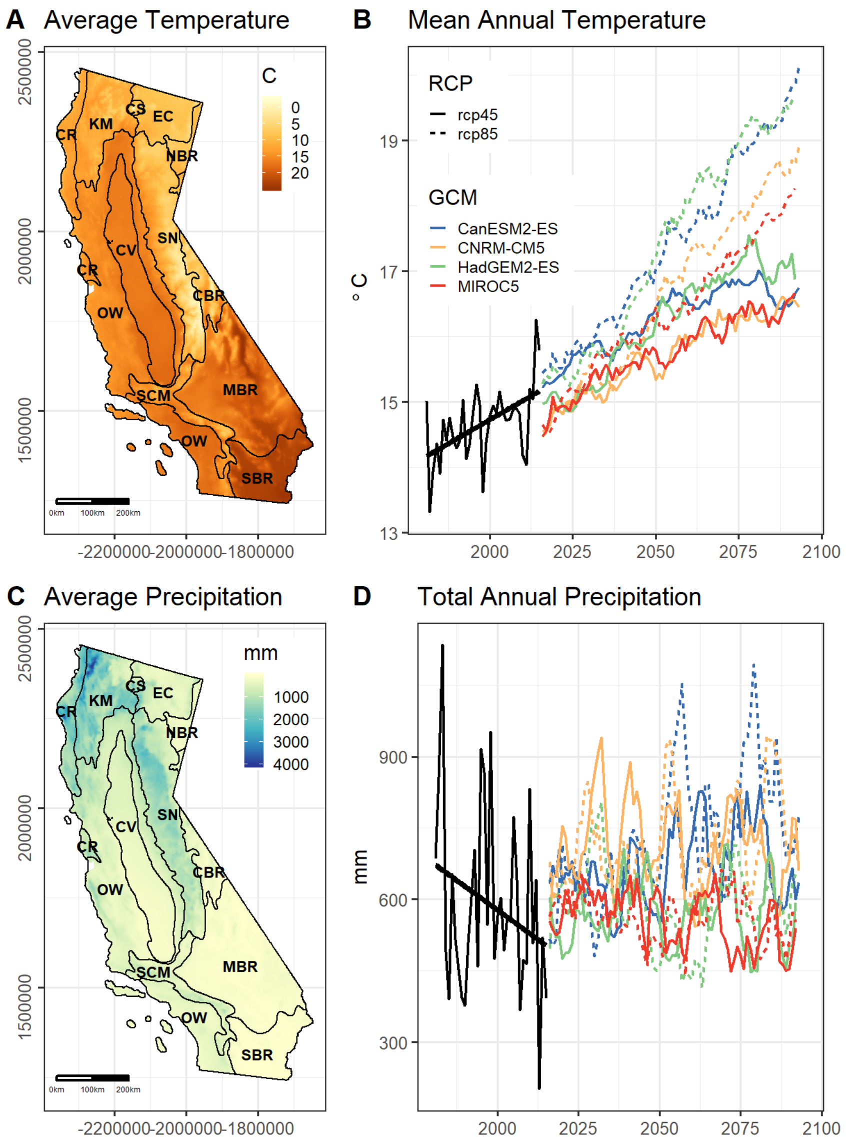

Figure 5: Maps show a) historical average annual temperature and c) precipitation based on 30-year climate normals. Plots show projected b) mean annual temperature and d) precipitation for California based on four climate models and two RCP scenarios from the LOCA down-scaled projections. Black lines show historical data based on PRISM. Projected data show the rolling 5-year average for temperature and the rolling 10-year average for precipitation.

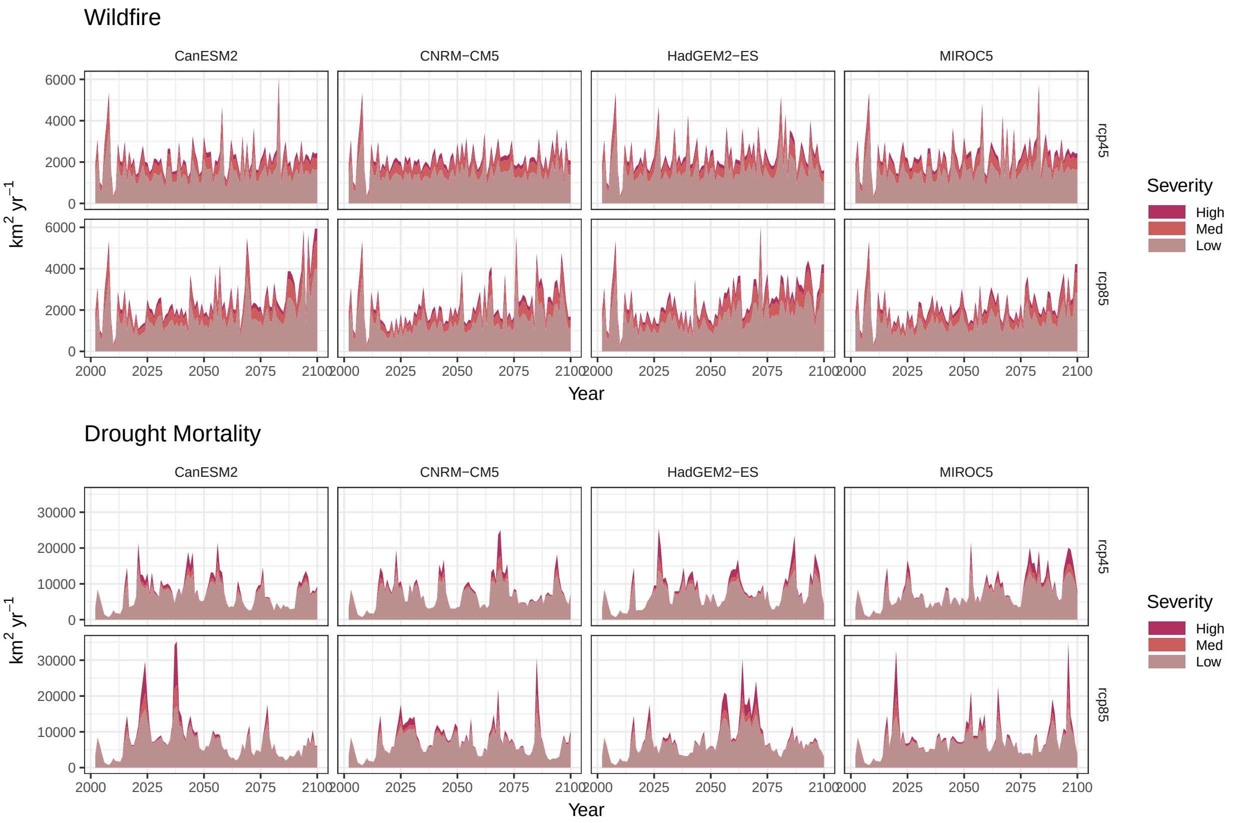

Figure 6: Mean annual estimated transition area for wildfire (top) and drought-induced tree mortality (bottom). Estimates are shown for each climate model (GCM; columns) and radiative forcing scenario (RCP; rows).

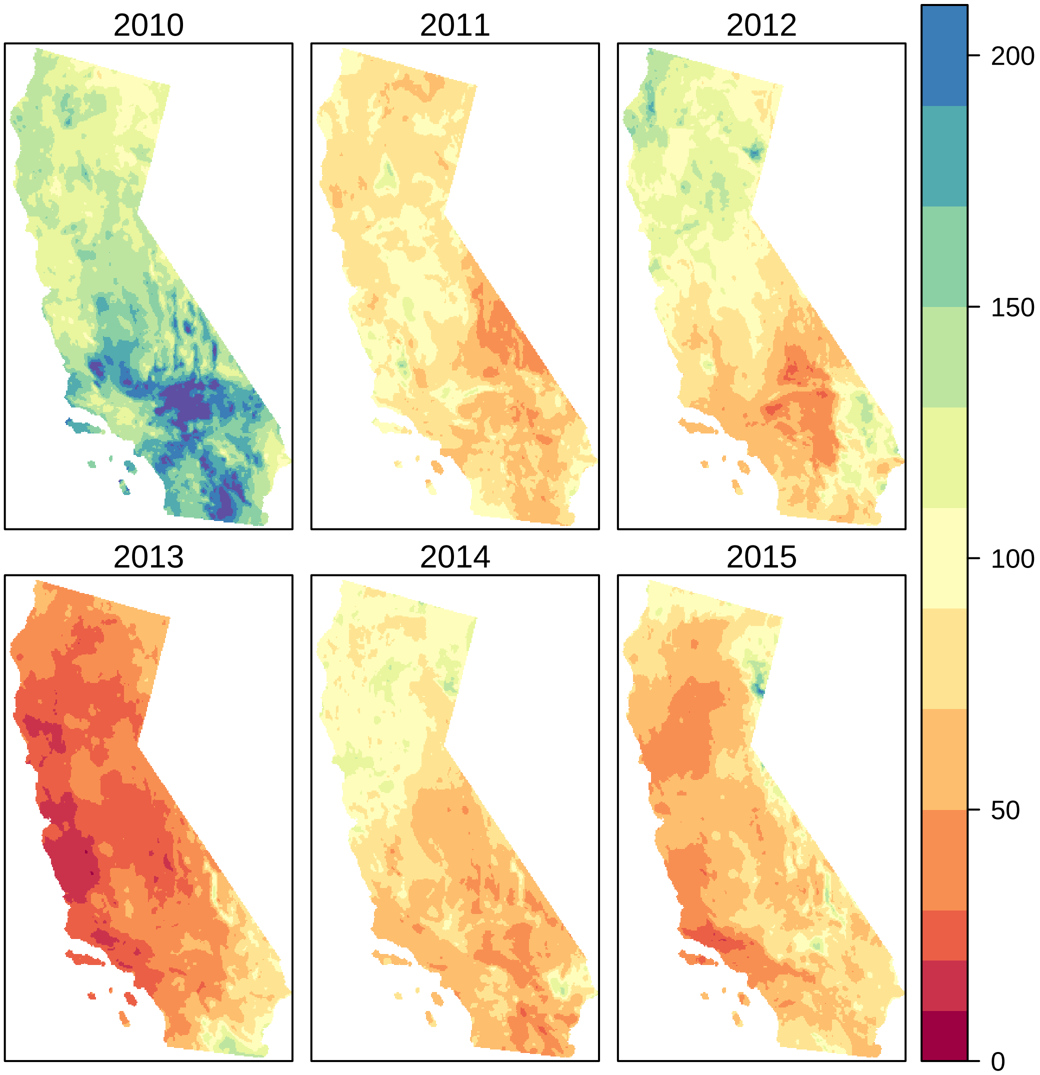

Figure 7: Annual precipitation anomaly for 2010-2015. Base period is from 1981-2010. Data are from the PRISM Climate Group.

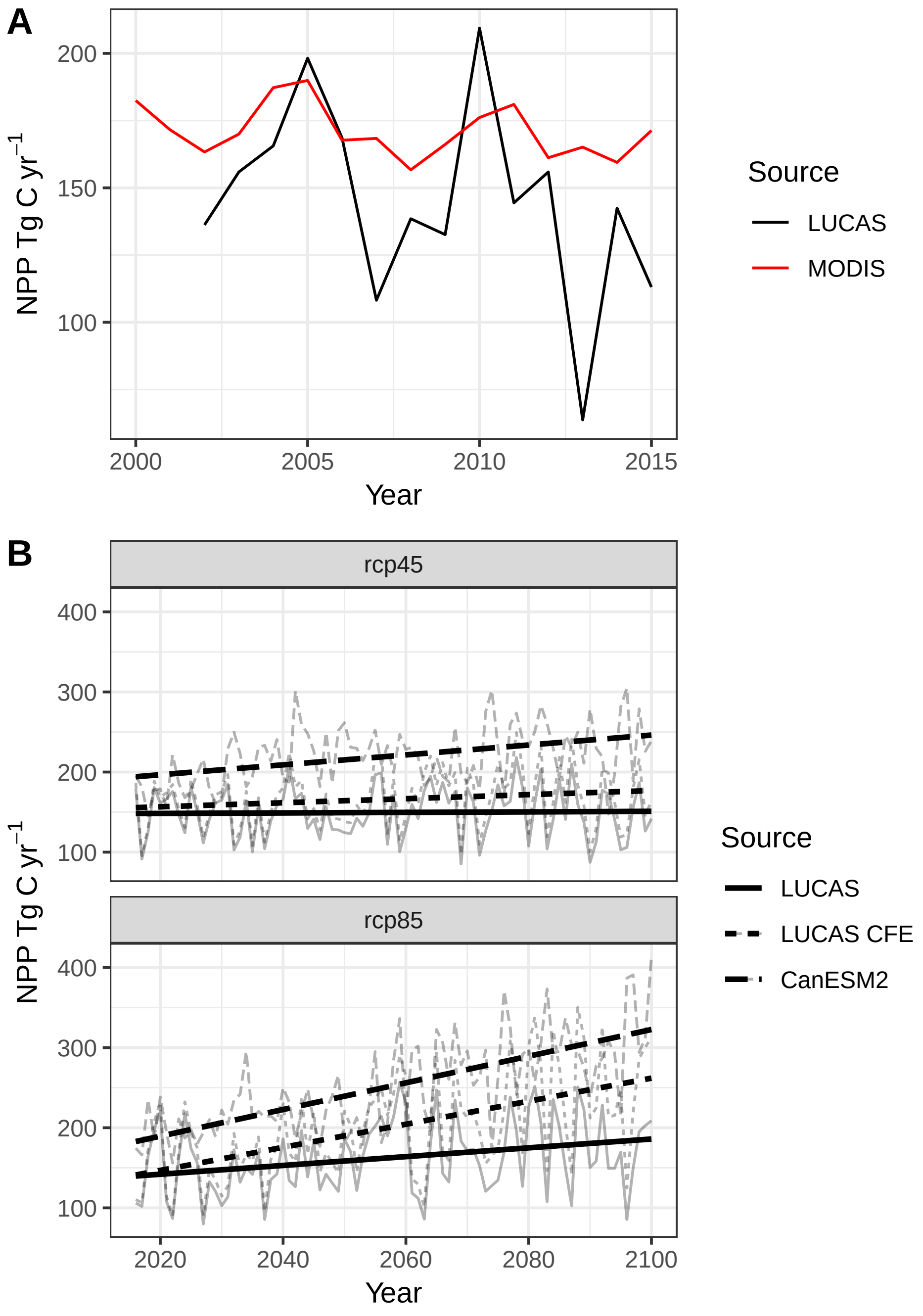

Figure 8: Comparison of net primary productivity over the historical and future periods. Panel A shows a historical comparison (2002-2015) between this study and estimates from MODIS. Panel B shows a comparison over future years (2016-2100) shows estimates from this study, compared with estimaates from an Earth System Model for both RCP scenarios. Also included are estimates incorporating a High CFE.

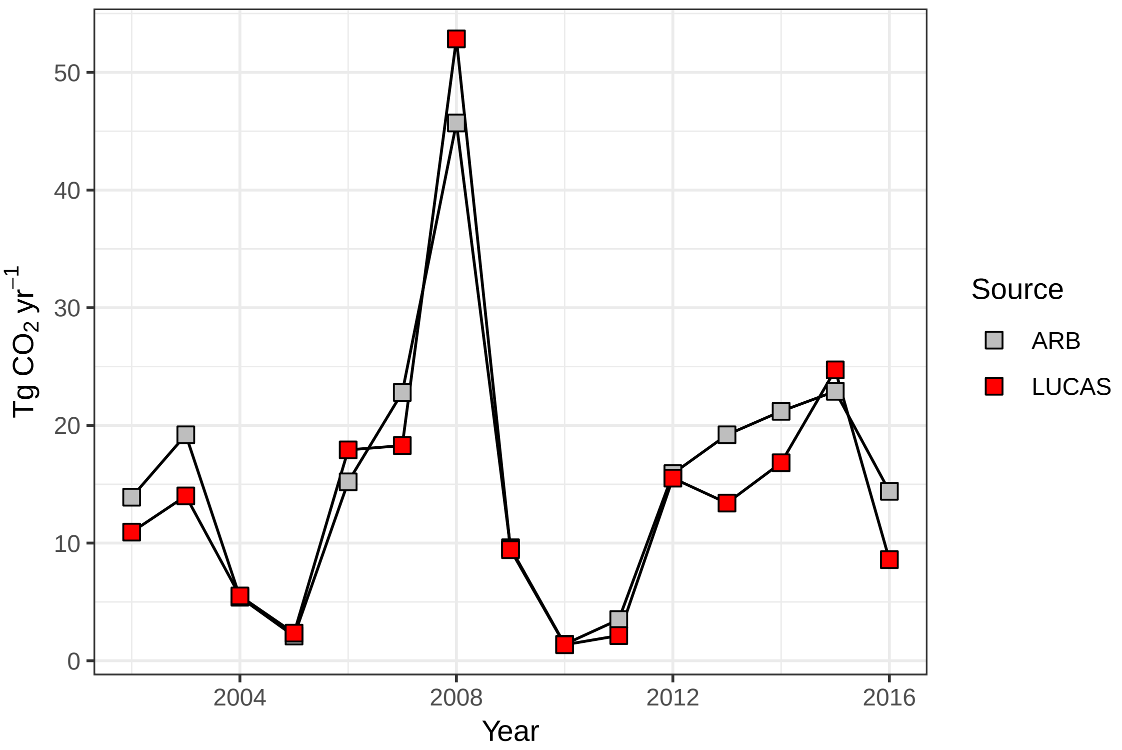

Figure 9: Comparison of modeled estimates of wildfire emissions in California from this study (LUCAS) and the California Air Resources Board (ARB).

Table 1: Comparison of carbon stocks from this study to other recent studies. Summaries of each study were based on resampling raster carbon stock maps to match the spatial extent, resolution, and projection of this study. Blackard; Estimates include above-ground live biomass carbon only. Gonzalez; Wildlands follow IPCC classification and include forestland (including shrubland and woodland), grassland, wetlands, and other land, excluding cropland and settlements. Estimates include above-ground live biomass carbon only. Kellendorfer; Estimates include above-ground live biomass carbon only. SSURGO; All valid cell values contained in the SSURGO map were included and were based on estimates to 2-meters depth. Wilson; Estimates of Live include above and below-ground live biomass carbon. DOM includes carbon stored in standing deadwood, down deadwood, and litter pools. This Study; Includes estimates for all lands classified as forest, grassland, shrubland, and agriculture (annual and perennial) but excludes wetlands and settlements. Live estimates include above and below ground carbon. SOC includes carbon stored up to 2-meters depth.

		Tg C		
Source	Ecosystems	DOM	Live	SOC
Blackard	Forest	—	1065.2	—
Gonzalez 2001	Wildlands	—	918.1	—
Gonzalez 2010	Wildlands	—	849.3	—
Kellendorfer	Forest	—	894.7	—
SSURGO	Wildlands + Ag	—	—	1851.5
This Study 2001	Wildlands + Ag	375.9	1804.3	2643.0
This Study 2010	Wildlands + Ag	389.2	1799.3	2604.4
Wilson	Forest	619.3	1113.4	538.2

References

Badgley, G., Field, C. B., & Berry, J. A. (2017). Canopy near-infrared reflectance and terrestrial photosynthesis. *Science Advances*, *3*(3), e1602244.

Boryan, C., Yang, Z., Mueller, R., & Craig, M. (2011). Monitoring us agriculture: The us department of agriculture, national agricultural statistics service, cropland data layer program. *Geocarto International*, *26*(5), 341–358.

CalFIRE. (2016). Fire Perimeters. Retrieved from http://frap.cdf.ca.gov/data/frapgisdata/select.asp

California Department of Water Resources. (2017). Statewide crop mapping 2014. Retrieved from https://gis.water.ca.gov/app/CADWRLandUseViewer/

Candel, A., Parmar, V., LeDell, E., & Arora, A. (2016). Deep learning with h2o. *H2O. Ai Inc.*

Chander, G., Markham, B. L., & Helder, D. L. (2009). Summary of current radiometric calibration coefficients for landsat mss, tm, etm+, and eo-1 ali sensors. *Remote Sensing of Environment*, *113*(5), 893–903.

Cleveland, C. C., Taylor, P., Chadwick, K. D., Dahlin, K., Doughty, C. E., Malhi, Y., … Townsend, A. R. (2015). A comparison of plot-based satellite and earth system model estimates of tropical forest net primary production. *Global Biogeochemical Cycles*, *29*(5), 626–644.

Del Grosso, S., Parton, W., Stohlgren, T., Zheng, D., Bachelet, D., Prince, S., … Olson, R. (2008). Global potential net primary production predicted from vegetation class, precipitation, and temperature. *Ecology*, *89*(8), 2117–2126.

Eidenshink, J., Schwind, B., Brewer, K., Zhu, Z.-L., Quayle, B., & Howard, S. (2007). A project for monitoring trends in burn severity. *Fire Ecology*, *3*(1), 3–21.

Gesch, D., Oimoen, M., Greenlee, S., Nelson, C., Steuck, M., & Tyler, D. (2002). The national elevation dataset. *Photogrammetric Engineering and Remote Sensing*, *68*(1), 5–32.

Goward, S., Huang, C., Masek, J., Cohen, W., Moisen, G., & Schleeweis, K. (2012). NACP north american forest dynamics project: Forest disturbance and regrowth data. *ORNL DAAC.*

Hengl, T., Jesus, J. M. de, Heuvelink, G. B., Gonzalez, M. R., Kilibarda, M., Blagotić, A., … others. (2017). SoilGrids250m: Global gridded soil information based on machine learning. *PLoS One*, *12*(2), e0169748.

Hengl, T., Jesus, J. M. de, MacMillan, R. A., Batjes, N. H., Heuvelink, G. B., Ribeiro, E., … others. (2014). SoilGrids1km—global soil information based on automated mapping. *PloS One*, *9*(8), e105992.

Homer, C., Dewitz, J., Yang, L., Jin, S., Danielson, P., Xian, G., … Megown, K. (2015). Completion of the 2011 national land cover database for the conterminous united states–representing a decade of land cover change information. *Photogrammetric Engineering & Remote Sensing*, *81*(5), 345–354.

Kurz, W., Dymond, C., White, T., Stinson, G., Shaw, C., Rampley, G., … others. (2009). CBM-cfs3: A model of carbon-dynamics in forestry and land-use change implementing ipcc standards. *Ecological Modelling*, *220*(4), 480–504.

Landscape Ecology, Modeling, Mapping, and Analysis (LEMMA). (2018). GNN Structure (Species-Size) Maps. Retrieved from https://lemma.forestry.oregonstate.edu/data/structure-maps

Moore, J., McAfee, L., & Iaccarino, J. (2018). 2017 aerial survey results: California. *United States Department of Agriculture (USDA)*, 1–18. Retrieved from https://www.fs.usda.gov/Internet/FSE_DOCUMENTS/fseprd583288.pdf

Obermeier, W., Lehnert, L., Kammann, C., Müller, C., Grünhage, L., Luterbacher, J., … others. (2017). Reduced co2 fertilization effect in temperate c3 grasslands under more extreme weather conditions. *Nature Climate Change*, *7*(2), 137.

Ohmann, J. L., Gregory, M. J., Henderson, E. B., & Roberts, H. M. (2011). Mapping gradients of community composition with nearest-neighbour imputation: Extending plot data for landscape analysis. *Journal of Vegetation Science*, *22*(4), 660–676.

Pierce, D. W., Cayan, D. R., & Thrasher, B. L. (2014). Statistical downscaling using localized constructed analogs (loca). *Journal of Hydrometeorology*, *15*(6), 2558–2585.

Pries, C. E. H., Castanha, C., Porras, R., & Torn, M. (2017). The whole-soil carbon flux in response to warming. *Science*, *355*(6332), 1420–1423.

PRISM Climate Group, Oregon State University. (2016). PRISM climate data. Retrieved from http://prism.oregonstate.edu

Ramcharan, A., Hengl, T., Nauman, T., Brungard, C., Waltman, S., Wills, S., & Thompson, J. (2018). Soil property and class maps of the conterminous united states at 100-meter spatial resolution. *Soil Science Society of America Journal*.

Reich, P. B., Hobbie, S. E., & Lee, T. D. (2014). Plant growth enhancement by elevated co2 eliminated by joint water and nitrogen limitation. *Nature Geoscience*, *7*(12), 920.

Sleeter, B. M., Liu, J., Daniel, C., Rayfield, B., Sherba, J., Hawbaker, T. J., … Loveland, T. R. (2018). Effects of contemporary land-use and land-cover change on the carbon balance of terrestrial ecosystems in the united states. *Environmental Research Letters*, *13*(4), 045006.

Sleeter, B. M., Wilson, T. S., Sharygin, E., & Sherba, J. T. (2017). Future scenarios of land change based on empirical data and demographic trends. *Earth's Future*, *5*(11), 1068–1083.

Smith, W. K., Reed, S. C., Cleveland, C. C., Ballantyne, A. P., Anderegg, W. R., Wieder, W. R., … Running, S. W. (2016). Large divergence of satellite and earth system model estimates of global terrestrial co 2 fertilization. *Nature Climate Change*, *6*(3), 306.

Stephens, S. L., Collins, B. M., Fettig, C. J., Finney, M. A., Hoffman, C. M., Knapp, E. E., … Wayman, R. B. (2018). Drought, tree mortality, and wildfire in forests adapted to frequent fire. *BioScience*, *68*(2), 77–88.

Tifafi, M., Guenet, B., & Hatté, C. (2018). Large differences in global and regional total soil carbon stock estimates based on soilgrids, hwsd, and ncscd: Intercomparison and evaluation based on field data from usa, england, wales, and france. *Global Biogeochemical Cycles*, *32*(1), 42–56.

Vicente-Serrano, S. M., Beguera, S., & Lopez-Moreno, J. I. (2010). A multiscalar drought index sensitive to global warming: The standardized precipitation evapotranspiration index. *Journal of Climate*, *23*(7), 1696–1718.

Westerling, A. L. (2018). Wildfire simulations for california's fourth climate change assessment: Projecting changes in extreme wildfire events with a warming climate. Re-

trieved from http://www.climateassessment.ca.gov/techreports/docs/20180827-Projections_CCCA4-CEC-2018-014.pdf
